# Species life‐history strategies affect population responses to temperature and land‐cover changes

**DOI:** 10.1111/gcb.16454

**Published:** 2022-10-17

**Authors:** Gonzalo Albaladejo‐Robles, Monika Böhm, Tim Newbold

**Affiliations:** ^1^ Centre for Biodiversity and Environment Research, Department of Genetics, Evolution and Environment University College London London UK; ^2^ Institute of Zoology Zoological Society of London London UK; ^3^ Global Center for Species Survival Indianapolis Indiana USA

**Keywords:** climate, land‐use change, life‐histories, living planet index, macroecology, population trends, terrestrial vertebrates

## Abstract

Human‐induced environmental changes have a direct impact on species populations, with some species experiencing declines while others display population growth. Understanding why and how species populations respond differently to environmental changes is fundamental to mitigate and predict future biodiversity changes. Theoretically, species life‐history strategies are key determinants shaping the response of populations to environmental impacts. Despite this, the association between species life histories and the response of populations to environmental changes has not been tested. In this study, we analysed the effects of recent land‐cover and temperature changes on rates of population change of 1,072 populations recorded in the Living Planet Database. We selected populations with at least 5 yearly consecutive records (after imputation of missing population estimates) between 1992 and 2016, and for which we achieved high population imputation accuracy (in the cases where missing values had to be imputed). These populations were distributed across 553 different locations and included 461 terrestrial amniote vertebrate species (273 birds, 137 mammals, and 51 reptiles) with different life‐history strategies. We showed that populations of fast‐lived species inhabiting areas that have experienced recent expansion of cropland or bare soil present positive populations trends on average, whereas slow‐lived species display negative population trends. Although these findings support previous hypotheses that fast‐lived species are better adapted to recover their populations after an environmental perturbation, the sensitivity analysis revealed that model outcomes are strongly influenced by the addition or exclusion of populations with extreme rates of change. Therefore, the results should be interpreted with caution. With climate and land‐use changes likely to increase in the future, establishing clear links between species characteristics and responses to these threats is fundamental for designing and conducting conservation actions. The results of this study can aid in evaluating population sensitivity, assessing the likely conservation status of species with poor data coverage, and predicting future scenarios of biodiversity change.

## Graphical abstract

In this work, we showed how fast‐lived species populations present positive population growth rates in areas that have experienced recent temperature and land‐use changes. Whereas populations of slow‐lived species showed the opposite response.

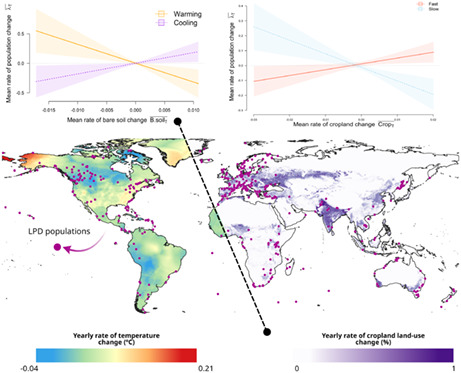

## 1 INTRODUCTION

Human‐driven environmental changes are driving rapid changes in biodiversity globally. The latest Living Planet Report estimated that monitored populations of the world's vertebrates have declined on average by 68% since 1970 (WWF, 2020), and that further decreases are expected into the future. Land‐use change has been identified as one of the main drivers of global biodiversity loss (Newbold et al., 2015; WWF, 2020), while the effects of climate change on biodiversity are likely to intensify in the next decades (Newbold, 2018). In this context, prioritization of especially sensitive biomes and species is essential to allocate conservation efforts and reduce biodiversity loss (Strassburg et al., 2020; Watson et al., 2020). However, to design long‐term effective conservation strategies, we first need to understand the factors that determine how species and communities respond to environmental changes (Keith et al., 2015).

It is known that climate warming affects species and communities, causing species range shifts (Parmesan & Yohe, 2003), phenological changes and asynchrony of biological processes (Donnelly et al., 2011), and species extinctions (Román‐Palacios & Wiens, 2020; Sinervo et al., 2010; Spooner et al., 2018). Similarly, land‐use changes cause habitat destruction and fragmentation (Daye & Healey, 2015), homogenization of communities (Gossner et al., 2016), and loss of species richness (Murphy & Romanuk, 2014). Furthermore, the interaction between climate warming and land‐use change can exacerbate these effects (Northrup et al., 2019). Predicting how species may respond to climate and land‐use changes is of paramount importance. Previous studies have shown that species with certain traits, such as large size or long generation length, are more sensitive than others (e.g. Laliberté et al., 2010; Pacifici et al., 2015, 2017). However, these patterns varied across taxonomic groups and traits, making extrapolations of species' responses to environmental changes challenging (Laliberté et al., 2010; Pacifici et al., 2015, 2017). It is necessary then to find a more generalized and taxon‐independent approach to predict responses to environmental changes.

Species life‐history strategies can serve as a proxy to evaluate species' extinction risk and sensitivity to environmental changes (Kosydar, 2014; Richards et al., 2021). Life‐history strategies are defined by the intrinsic trade‐offs between species traits related to ageing (e.g. longevity, growth rates, or maturity) and fecundity (e.g. litter/clutch size or frequency of reproduction) (Dobson & Oli, 2007, 2008). Depending on the values of these life‐history traits, species can be positioned along a continuum from fast‐ to slow‐lived species (Read & Harvey, 1989; Stearns, 1983a). Species located towards the faster end of the continuum (fast species hereafter) have higher fecundity and shorter lifespans. In contrast, species closer to the slower end of the continuum (slow species hereafter) display longer lifespans and lower fecundity.

Theoretically, fast and slow species have different population‐regulation mechanisms (MacArthur & Wilson, 1967). According to r/K‐selection theory, fast species, or r‐selected species, show density independent or stochastic mortality events, with their populations unable to reach the environmental carrying capacity (MacArthur & Wilson, 1967; Pianka, 1970). Owing to their rapid fluctuations, populations of fast species are, theoretically, adapted to recover their populations faster than slow species (Pianka, 1970). Conversely, slow species, or K‐selected species, show density‐dependent mortality (MacArthur & Wilson, 1963; Pianka, 1970). K‐selected species are adapted to maintain stable populations close to the carrying capacity of the environment, and take longer to reach this population level after a stochastic mortality event (Pianka, 1970). For this reason, slow species are usually considered more sensitive to environmental changes, and thus more likely to show population declines due to human impacts (e.g. Bird et al., 2020). Although these ideas are frequently used in ecology and conservation, the role of life‐history strategies in shaping the response of species' populations to environmental changes has not been empirically tested at a large scale (Stearns, 2000; Sutherland et al., 2013).

Life‐history strategies have been shown to correlate with species' responses to habitat heterogeneity and land‐use disturbance. Previous studies looking at traits related with ageing and fecundity have found that, in general, fast‐lived species were more likely to be present in more human‐impacted land‐use areas, whereas slow species thrive in less impacted habitats (Newbold et al., 2013). Fast and slow species also respond differently to climate change. Generally, species with fast life‐history traits tolerate and, in some cases benefit, from climate warming (Lehmann et al., 2020; Pacifici et al., 2017). In contrast, species with slow life‐history traits were more sensitive to climate warming (Pacifici et al., 2017; Richards et al., 2021). However, the role of species life‐history strategies in shaping population trend responses to environmental changes is still unclear.

Large databases of population trends, such as the Living Planet Database (LPD) which holds the data underlying the Living Planet Index (LPI), allow us to explore changes in vertebrate populations over time. The LPI was developed to measure the changing state of the world's biodiversity (WWF, 2020), and relies on time‐series data to calculate average rates of change in populations of terrestrial, freshwater, and marine vertebrate species. Although this index has been widely adopted to report on global (WWF, 2020) and local biodiversity changes (e.g. van Strien et al., 2016), the LPI is not free from bias or criticism. Studies analysing the robustness of the LPI have shown major changes in global populations trends depending on the inclusion or removal of a small fraction of populations with extreme trends (Leung et al., 2020; see also Loreau et al., 2022; Mehrabi & Naidoo, 2022, for an extended discussion). Similarly, random population fluctuations can compromise the accuracy of the LPI even if, on average, populations remain stable, especially when populations are small (Buschke et al., 2021). Furthermore, the underlying time‐series data disproportionately represent osseous fishes and birds. These two groups of vertebrates are represented by 1,408 and 1,494 species, respectively, and account for more than 70% of the total populations in the LPD. Conversely, mammals and reptiles are represented for 636 and 227 species, forming 21% of populations in the LPD. Additionally, populations receiving conservation interventions are also overrepresented in the LPD (Murali et al., 2022). Some of the taxonomic and geographical bias has been addressed recently through the development of a weighted LPI (McRae et al., 2017). Most of these issues arise from a lack of population‐monitoring data, a limitation not restricted to the LPI (Hochkirch et al., 2021).

Population‐monitoring data for most species are still rare (e.g. Bland & Böhm, 2016), but their use in conservation is of paramount importance. For example, some criteria of the IUCN Red List of Threatened Species are based on population sizes (criteria C and D) and declines (criteria A and C) (IUCN, 2021). In this context of data deficiency and increasing need for biodiversity conservation, establishing clear links between species life‐history strategies and population trends under environmental changes can help us to (1) identify groups of species whose life‐history strategies make then more likely to be sensitive to climate and land‐use changes; (2) establish a more general or extrapolatable relationship between species traits and responses to environmental changes; and (3) better predict how poorly monitored species would respond to future climate and land‐use changes.

**FIGURE 1:**
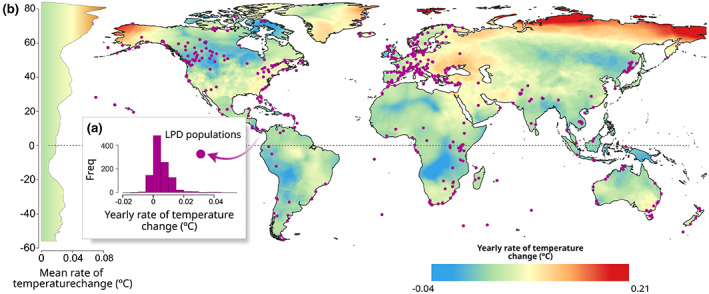
Distribution of the populations selected for the analysis (purple points), and the average rates of climate warming (annual mean temperature) for the period between 1992 and 2018 (base layer). Panel a shows the distribution of yearly rates of temperature change (see Climate and land‐use data) for the populations included in the analysis. Panel b shows the latitudinal mean yearly rate of warming. Yearly rates of warming were calculated using the Climatic Research Unit gridded Time Series (CRUTS) Version 4.04 dataset (Harris et al., 2020). The outline world map was extracted from the Database of Global Administrative Areas (GADM) (https://gadm.org/). All the spatial information was projected using the WGS84 coordinate reference system (EPSG: 4326).

In this study, we explore how populations of terrestrial amniote vertebrate species with different life‐history strategies respond to recent climate and land‐use changes. To do this, we use the population trends reported in the public LPD. We extracted mean annual rates of population change, land‐cover and temperature change for 1,072 populations of 461 species (273 birds, 137 mammals, and 51 reptile species), distributed across 553 locations globally (Figure 1). We hypothesized that species with different life‐history strategies will show distinct population trends depending on recent climate and land‐use changes where they occur. Fast‐lived species are expected to be better adapted to recover after environmental perturbations. Therefore, on average, we expect fast species to show less negative population trends than other species, and for a greater proportion of species to show positive trends, in more human‐disturbed habitats, for example areas where croplands or urban areas are expanding. Conversely, we expect a greater proportion of slow species, under the same conditions, to present declining populations, and for average population trends to be more negative. We expect a similar response to climate change, with fast‐lived species populations responding more positively to temperature warming compared to slow‐lived species.

## 2 MATERIALS AND METHODS

### 2.1 Species populations trends

We extracted population trends from the public LPD (LPI, 2016) (https://livingplanetindex.org/home/index). This database contains time‐series information for 15,349 populations worldwide, of 4,182 different species of terrestrial, freshwater, and marine vertebrates. This dataset contains geographical information regarding the locations of monitored populations, but it does not include information regarding the geographical extent over which species are monitored. The LPD encompasses more than six decades of species monitoring data, from 1950 to 2018 (Collen et al., 2009; McRae et al., 2017). Time series in the LPD were collected from scientific studies, online databases, and the grey literature (Collen et al., 2009), and must meet a series of criteria to be included in the LPD (see Loh et al., 2005 for a detailed list). Importantly, time series must have at least two time points, and the method used to calculate the species population size or abundance must be compatible/comparable (e.g. count, density, and abundance).

The choice to focus on amniote species was based on the reproductive similarities between these groups. We considered mammals, reptiles, and birds more feasible to group and compare than, for example, birds and amphibians. The public LPD contains data on 8,112 populations of terrestrial amniotes (54% of the total LPD data) from 2,357 species (1,494 birds, 636 mammals, and 227 reptiles). To match the periods covered by our environmental data (see **Climate and land‐use data** for more details), only populations with at least three data points between 1992 and 2018 and a known location (latitude and longitude) were included in the analysis.

Overall, 2,327 populations in the LPD met our criteria. For this subset, we calculated rates of population change following the approach used for the LPI (Collen et al., 2009). Population records were log_10_ transformed (Collen et al., 2009). 11% of populations contained zeros, which were replaced by 1% of the mean of non‐zero abundances recorded across the population time series (Collen et al., 2009). To calculate the annual rate of population change, for each year and population, we required uninterrupted population estimates (i.e. for every consecutive year) (Spooner et al., 2018). However, long‐term and continuous population time series are still rare (Hochkirch et al., 2021), even in the LPD (http://stats.livingplanetindex.org/). Approximately 40% of the 2,327 populations contained missing observations. Therefore, we implemented imputation methods to fill those gaps and obtain continuous time series. We followed the imputation approach used for the LPI (Collen et al., 2009), adopting two methods to impute population observations depending on the number of real records available for each population time series: (1) a linear‐regression interpolation was implemented when fewer than six real observations were available (Loh et al., 2005) or (2) for time series with six or more records, we implemented generalized additive models (GAMs). Linear regressions and GAMs were fitted for each time series using the population estimates as the response variable and year as the explanatory variable. In the case of the simple regression models, only linear relationships were considered due to the low number of observations. In the GAMs, year was fitted with a smooth parameter, in the form of a penalized regression spline, of dimension equal to the length of the population time‐series divided by two (Collen et al., 2009; McRae et al., 2017). Fitted GAMs and linear models were later used to impute missing values of their corresponding time series. We did not impute values outside of the range (first to last year) of each time series. Model fit for both linear regression and GAMs was evaluated using *R* ^*2*^ values. Since we wanted to include only populations with low imputation uncertainty in our analysis, we selected those populations with an *R* ^*2*^ of at least 0.5 (following Spooner et al., 2018).

Another potential source of uncertainty arises from variability in time‐series length. Short time‐series are more likely to return false negative/positive trends, or to miss real and statistically significant population trends (Wauchope et al., 2019). To reduce this uncertainty, we discarded all populations with fewer than five observations (real or imputed) (Spooner et al., 2018) between the years 1992 and 2018. We select this period to make the timespan covered by the LPD compatible with the land‐cover data (see **Climate and land‐use data** for more details). Although there is no standardized way to select the optimal length of a population time‐series (Wauchope et al., 2019), the approach we used here offers an adequate time‐series length while still having a large enough dataset for analysis.

We used the imputed and real values of the selected population time‐series (*n*_*t*_) to calculate the annual (log_10_) rate of population change ($\lambda_{T}$) for each year and population, as described in Equation (1). These values were then averaged to obtain the mean (log_10_) rate of population change for each population ($\bar{\lambda_{T}}$) (Equation 2):

(1) $$\lambda_{t}=\log_{10}\left(\frac{n_{t}}{n_{t-1}}\right)$$

(2) $$\bar{\lambda_{T}}=\frac{1}{T}\;\sum_{t=1}^{T}\lambda_{t}$$

where *n* represents real and imputed population measures, *t* represents the time/year at which the measure was taken/imputed, and *T* is the total number of years from the first to the last population estimates (Collen et al., 2009).

Our final dataset contained average population trend estimates for 1,072 populations. These populations were distributed in 553 different locations across the world, and represented 461 different species (273 birds, 137 mammals, and 51 reptiles) (Figure 1). On average, after imputation, our populations had a mean time‐series length of 10 ± 4.2 (standard deviation) records. Linear‐regression interpolations were performed using the R package *stats* Version 4.0.2 (R Core Team, 2021), and GAMs were fitted using the R package *mgcv* Version 1.8‐31 (Dunn & Smyth, 2018).

### 2.2 Climate and land‐use data

We extracted temperature values from the Climatic Research Unit gridded Time Series (CRUTS) Version 4.04 dataset (Harris et al., 2020). This dataset contains monthly measurements of land‐surface temperature at a grid resolution of 0.5° (≈55 km at the equator). To measure temperature warming, we used the monthly values of temperature to calculate the arithmetic mean annual temperature values for the years and locations of each population time‐series (Figure 1). Within each population time‐series, we fitted linear regression models using yearly mean temperature as the response variable and year as the only explanatory variable. The slope of these models was extracted and used as the average annual rate of temperature warming (∆T in Equation 3) for the time period and location of each of our populations. The strong inter‐annual fluctuations in temperature make it necessary to use a linear regression to capture overall trends over time (Bowler et al., 2020; Spooner et al., 2018).

We gathered land‐cover information from the European Space Agency Climate Change Initiative Land Cover Project (ESA CCI LCP, http://www.esa‐landcover‐cci.org) Version 2.0.7 (ESA‐LC hereafter). This dataset contains global time series of land‐cover data at a grid resolution of 300 m from 1992 to 2018. The ESA‐LC consists of a map, in which each pixel on the grid is classified into one of 36 discrete land‐cover types. To extract land‐cover trends, we transformed the discrete categorization of land cover into the percent coverage of broader land‐cover categories (Li et al., 2018). For this transformation, we used a cross‐walking table between the ESA‐LC classes and 14 simplified land‐cover classes (Li et al., 2018): broadleaf evergreen trees, broadleaf deciduous trees, needleleaf evergreen trees, needleleaf deciduous trees, broadleaf evergreen shrubs, broadleaf deciduous shrubs, needleleaf evergreen shrubs, needleleaf deciduous shrubs, natural grass, bare‐soil, cropland, snow/ice, urban, and water (see Table S6 for the cross‐walking table). Following Li et al. (2018), we grouped all the tree and shrub land‐use classes into two final classes: forest trees and shrubs, resulting in 8 final land‐cover classes for our analysis. We then extracted the annual percentage change in each land‐cover type for each time period encompassing each population time series within a 1‐km‐radius buffer around each population's location. We used this buffer because the LPD does not provide information about the spatial range occupied by each species populations. Instead, population locations in the LPD are represented by discrete coordinates that can represent sample stations or the centroid of a grid or polygon of the surveyed area. Furthermore, this buffer covered the distances and areas usually covered during biodiversity monitoring (e.g. Nalwanga et al., 2012; Newson et al., 2008); thus, we were likely to include the habitats in which species were recorded. Finally, we calculated the mean annual rate of change of each of the land‐cover classes for each population time‐series (${\bar{\mathrm{Lu}1}}_{T}.\ldots +{\bar{\mathrm{LuN}}}_{T}$, in Equation 3) (Li et al., 2018). We did not use a linear regression for land‐use changes, as we did for temperature changes, because land use in any one location tends to change in a simple directional fashion, and does not show strong inter‐annual fluctuations.

All the spatial information was processed in R Version 4.0.2 (R Core Team, 2021) using the packages *raster* Version 3.3‐13 (Hijmans, 2020), *maptools* Version 1.0‐2 (Bivand & Lewin‐Koh, 2020) and *rgdal* Version 1.5‐16 (Bivand & Keitt, 2020).

### 2.3 Species traits and life‐history strategies

To describe general life‐history strategies for the different species and taxa, we selected five traits for which information was widely available for all the classes included in the analysis: (i) maximum longevity, in years (longevity); (ii) age of sexual maturity, in years (maturity); (iii) the yearly number of reproductive events (reproductive events); (vi) number of offspring per reproductive event (offspring); and (vi) body mass, in grams. We compiled this data from 24 open‐access datasets and published scientific papers (Table S1). This resulted in a final dataset that included partial and complete trait data for more than 21,000 species of terrestrial vertebrates. As a result of this trait compilation, and since the studies used to retrieve the data encompass several decades, there was not a cohesive taxonomy across them. For this reason, a taxonomic and synonym resolution (see Taxonomic resolution and Supplementary Materials S1 for more details) was needed before all the trait data could be combined into a single dataset.

After the taxonomic and synonym resolution, the dataset contained more than 21,000 unique species. For 87% of those species, we found trait records in multiple datasets. In these cases, when multiple estimates of life‐history traits for the same species were available within the same or across different datasets, values were log‐transformed, and the arithmetic mean calculated. The mean estimate was then back transformed and incorporated into the final trait dataset (for details, see Supplementary Materials S2). After the data aggregation, we found complete trait information for 82% of the species present in our subset of the LPD database. To get complete trait data for our target species, we ran a phylogenetic imputation analysis using a random‐forest algorithm (Penone et al., 2014). For this analysis, we used the first 10 eigenvectors derived from a synthetic phylogenetic tree built using the TimeTree interface (Hedges et al., 2015). Since we did not find phylogenetic information for all species, we also added taxonomic class, order, and family, as a proxy for kinship (see also Supplementary Materials S3). The combination of phylogeny and taxonomic information improved imputation accuracy (Figure S1). To further increase imputation accuracy, we used a sub‐sample of the trait data, selecting all the species for which we had data for at least three of the five traits. By combining this subset of species with our target species, we increased the amount of information available to the random‐forest algorithm and reduced imputation errors (Figure S1 and S2). As a result of this process, we obtained complete life‐history trait information for 9,618 species of terrestrial amniotes (4,638 birds, 2,765 mammals, and 2,215 reptiles) (Figures S3 and S4).

To describe the life‐history strategies of the different species, we classified them using the fast–slow continuum (Dobson & Oli, 2008; Healy et al., 2019; Stearns, 1983b). The fast–slow continuum represents the trade‐offs between fecundity and aging. Before traits were used to define species life‐histories, the influence of body mass and kinship was removed by regressing trait values against body mass using linear mixed‐effects models (LMMs) (Supplementary Materials S4). Each life‐history trait was log‐transformed and regressed against log‐transformed body mass, with higher taxonomic information, to the level of class, included as a nested random intercept (i.e. family nested within order, nested within class) (for details, see Supplementary Materials S4). Residuals of these models were used as log‐transformed and adjusted life‐history traits, and combined into a principal component analysis (PCA) to characterize species life‐history strategies (Supplementary Materials S5).

The first three axes of this PCA are assumed to describe species' life‐history strategies and explained more than 80% of the observed life‐history trait variation (Table S3). The first axis (PC1) reflects the expected trade‐offs between longevity and fecundity, and therefore was used to represent the fast‐slow continuum (Table S3). Low score values of PC1 corresponded with species with delayed sexual maturity, longer lifespans, and lower sexual productivity, that is, slow species. In contrast, species with high score values are characterized by shorter lifespans, earlier sexual maturity, and higher reproductive productivity, that is, fast species (details see Supplementary Materials S4).

Imputations were performed using the R package *missForest* Version 1.4 (Stekhoven et al., 2012) (for details, see Supplementary Materials S3). LMMs were fitted using the R package nlme Version 3.1‐157 (Pinheiro et al., 2021) and PCA was performed using the R package *ade4* Version 1.7‐15 (Bougeard & Dray, 2018) (for details, see Supplementary Materials S4).

### 2.4 Taxonomic resolution

Both the LPD and the life‐history dataset are the result of data compilation and aggregation of many underlying studies, spanning several decades. A consequence of this is that species taxonomy differs across and within datasets. Since we rely on the species binomial names to match each species with its corresponding life‐history strategy and population trend, we needed to ensure that taxonomy was as consistent as possible. To resolve possible taxonomic mismatches, we performed a taxonomic name and synonym resolution (Cooke et al., 2019; Etard et al., 2020). To update and correct the taxonomy as much as possible, we first checked species for typographical errors using the R package *taxize* Version 0.9.95 (Chamberlain et al., 2020). Once original binomial names were corrected, these were passed through the online repositories of the International Union for Conservation of Nature Red List of Threatened Species (IUCN Red List, https://www.iucnredlist.org/ Version 2021‐1) and the Integrated Taxonomic Information System (ITIS, https://www.itis.gov/, as downloaded on 12^th^ September 2021). If the species was present in these repositories, the matched scientific name was retrieved along with all its associated synonyms and higher‐level taxonomic information. Wherever possible, we chose our matching binomial and taxonomic classification from the IUCN Red List. If a species binomial name was not recorded in the IUCN Red List, we used the binomial name and taxonomy retrieved from ITIS to match our datasets.

When all available taxonomic information was retrieved, a unified taxonomic table was created containing the species name as it appeared in the original datasets, the corrected binomial name, a unique matching binomial name for each species, a list of potential synonyms, and the higher taxonomic classification linked with the matching binomial name. We used this taxonomic table to unify binomial species names and higher taxonomy across the life‐history database and the LPD. Once these fields were corrected, and all potential synonyms were resolved, the LPD and estimates of life‐history strategies were finally merged.

### 2.5 Statistical analysis

The spatial distribution of population locations can lead to variations in population trends among locations and species that is unrelated to climate and land‐cover change. To consider these possible effects, we included site and species identity as random intercepts in LMMs. To build the initial statistical model (Equation 3), we used the mean rate of log_10_‐transformed population change ($\bar{\lambda_{T}}$) as our response variable, with the rate of climate warming (∆T), the mean rates of change for our land‐cover types (${\bar{\mathrm{LuN}}}_{t}$), and species position along the fast–slow continuum (PC1) as fixed effects, along with all their two‐ and three‐way interactions (Equation 3). We built this initial model to consider all the possible interactions between rates of land‐use change, temperature change, and species life‐history strategies. We considered these terms to be important determinants of species populations rates. This initial model included a total of 28 different terms (we did not consider interactions between the cover of different land‐use types). Final model selection was performed using a backward stepwise model selection based on Akaike information criterion corrected (AICc) for small samples, since our ratio of sample to model terms is lower than 40 values. In this approach, all model terms are initially included in the model. Terms that contribute to increase the overall AICc value of the model were discarded one at a time and the new model re‐evaluated (Yamashita et al., 2007). This process was repeated until the model with the lowest AICc was found (Yamashita et al., 2007).

(3) $$\begin{array}{c} \bar{\lambda_{T}}\sim ∆T+\mathrm{PC}1+{\bar{\mathrm{Lu}1}}_{T}.\ldots \\ +{\bar{\mathrm{LuN}}}_{T}+∆T:\mathrm{PC}1+{\bar{\mathrm{Lu}1}}_{T}:\mathrm{PC}1+\ldots \\ +{\bar{\mathrm{LuN}}}_{T}:\mathrm{PC}1+{\bar{\mathrm{Lu}1}}_{T}:∆T+\ldots \\ +{\bar{\mathrm{LuN}}}_{T}:∆T+{\bar{\mathrm{Lu}1}}_{T}:\mathrm{PC}1:∆T+\ldots \\ +{\bar{\mathrm{LuN}}}_{T}:\mathrm{PC}1:∆T+\left(1|\text{Location}\right)+\left(1|\text{Species}\right) \end{array}$$

To evaluate the robustness of our results, we ran two different sensitivity analyses to investigate the effects of data quality and population selection on the results. To assess the effects of data quality, we resampled the LPD data using different *R* ^*2*^ thresholds. Lower *R* ^2^ values are associated with poor population size imputation performance and therefore less accurate estimations of population trends. Conversely, high imputation accuracy is associated with higher *R* ^2^ values, and imputed values closer to real population values. Additionally, by changing the *R* ^*2*^ threshold from 0.5 (as used for the main results) to 0.3, 0.7, and 0.9, we modified the number of populations included in the model (1,108 for a threshold of 0.3, 996 for a threshold of 0.7, and 915 for a threshold of 0.9) as well as the quality/uncertainty of the data. To assess the effects of population selection on our results, we ran a second analysis in which we selectively removed populations with extreme values of $\bar{\lambda_{T}}$ (similar to Leung et al., 2020 and Murali et al., 2022). In our case, we created three different datasets by (1) removing populations below the 2.5^th^ percentile of $\bar{\lambda_{T}}$; (2) removing populations above the 97.5^th^ percentile of $\bar{\lambda_{T}}$; and (3) preserving only the populations between the 2.5^th^ and 97.5^th^ percentiles of $\bar{\lambda_{T}}$.

It is also possible for inaccuracies to be introduced by the use of imputed life‐history traits. However, due to the high accuracy of our imputation process (see Supplementary Materials S3), we considered these inaccuracies to be low relative to other forms of uncertainty.

The mixed‐effects models were fitted using restricted maximum likelihood, implemented in the R package *lme4* Version 1.1‐26 (Bates et al., 2015) and backward stepwise model selection was implemented using the step function of the R package *stats* Version 4.0.5 (R Core Team, 2021). Model fit was evaluated using conditional and marginal pseudo‐R^2^ values calculated using the R package *MuMin* Version 1.43.17 (Barton, 2020). Statistical significance of fixed effects from the final models was assessed using the Wald chi‐squared test, implemented using the R package *car* Version 3.0‐9 (Fox & Weisberg, 2019).

## 3 RESULTS

After model selection, our final model contained the following variables: rate of climate warming (∆T); species position on the fast–slow continuum (*PC1*); mean annual rate of cropland change (${\bar{\text{Crop}}}_{T}$), and its interaction with species life‐history position (${\bar{\text{Crop}}}_{T}:\mathrm{PC}1$); mean annual rate of bare‐soil change (${\bar{\text{Bare Soil}}}_{T}$), and its interaction with species life‐history position (${\bar{\text{Bare Soil}}}_{T}:\mathrm{PC}1$), and the interaction between mean annual rate of bare‐soil change and climate warming ($T∆:{\bar{\text{Bare Soil}}}_{T}$) (Equation 4).

(4) $$\begin{array}{c} \bar{\lambda_{T}}\sim ∆T+\mathrm{PC}1+{\bar{\text{Crop}}}_{T}+{\bar{\text{Bare Soil}}}_{T}\\ +{\bar{\text{Crop}}}_{T}:\mathrm{PC}1+{\bar{\text{Bare Soil}}}_{T}:\mathrm{PC}1+∆T:{\bar{\text{Bare Soil}}}_{T}\\ +\left(1|\text{Location}\right)+\left(1|\text{Species}\right) \end{array}$$

**FIGURE 2:**
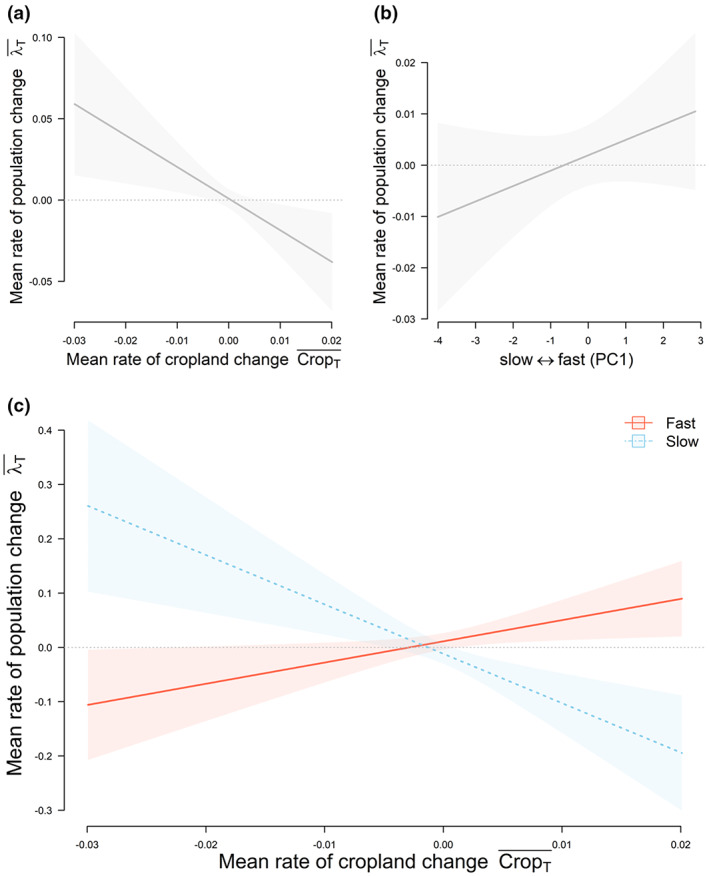
Responses of average population trends to: (a) rate of cropland change; (b) fast‐slow continuum; and (c) effects of the rate of cropland change for fast (red line/shaded area) and slow (blue dotted line/shaded area) species separately. Predicted responses in c are shown for the fastest and slowest‐lived species sampled in the LPD localities used in the analysis. Shaded areas represent the 95% confidence intervals around the fitted relationships. Model estimates, standard errors, and statistical significance of fixed effects are presented in Table 1.

**FIGURE 3:**
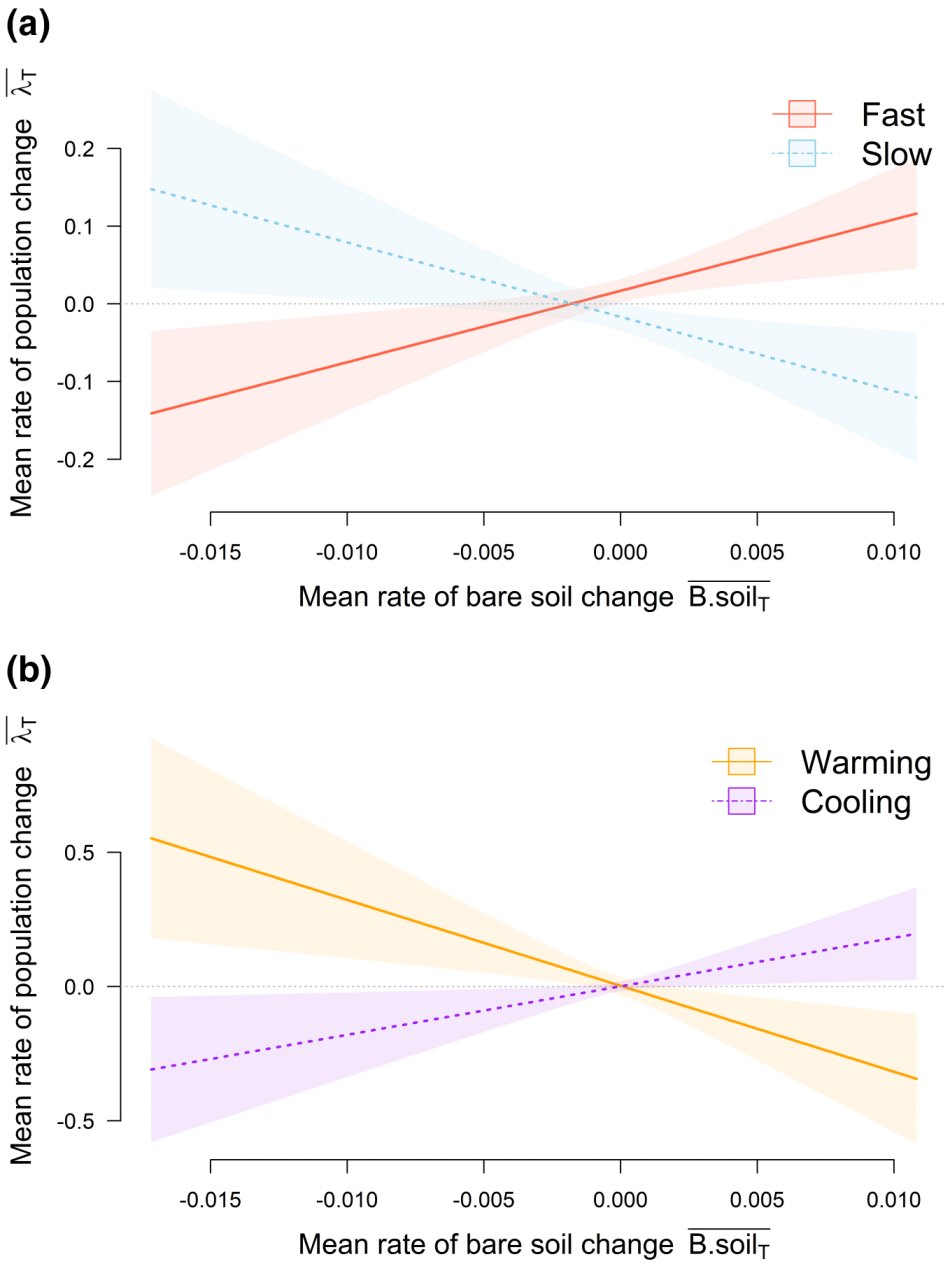
Responses of average population trends to: (a) rate of bare‐soil change for fast (red line/shaded area) and slow (blue dotted line/shaded area) species; and (b) rate of bare‐soil change for populations experiencing climate warming (gold line/shaded area) and climatic cooling (purple dotted line/shaded area). Predicted responses in (a) are shown for species with the highest and lowest values of the fast–slow continuum sampled in the LPD localities used in the analysis. Predicted responses in (b) are shown for the absolute maximum and minimum rates of temperature warming recorded within the sampled localities. Shaded areas represent the 95% confidence intervals around the fitted relationships. Model estimates, standard errors, and statistical significance of fixed effects are presented in Table 1.

Overall, populations of species inhabiting areas where cropland has expanded were decreasing, whereas populations inhabiting areas where cropland was contracting showed positive populations trends (Table 1; Figure 2a). Fast‐lived species tended to have more positive population trends than slow‐lived species (Table 1; Figure 2b). Population trends of fast and slow species also varied according to land‐cover changes (Table 1). Fast‐lived species inhabiting areas that had experienced recent cropland and bare‐soil expansion had positive population trends (Figures 2c and 3a). In contrast, populations of slow species inhabiting these areas tended to have negative population trends (Figure 2c and 3a).

**TABLE 1:** Estimates, standard errors, and results of an ANOVA type III Wald chi‐square test for the model of population trends

Term	Estimate	Std. error	*χ* ^2^	*p*
(Intercept)	0.0025	0.00281	0.871	.3506
∆T	0.0246	0.0024	0.0045	.9462
**PC1**	**0.0052**	**0.0023**	**5.0484**	**.0246**
${\bar{\text{Crop}}}_{T}$	**−1.4974**	**0.6943**	**4.6512**	**.031**
${\bar{\text{Bare Soil}}}_{T}$	5.1349	1.7809	0.6104	.088
**PC1**: ${\bar{\text{Crop}}}_{T}$	**1.896**	**0.6017**	**9.9275**	**.0016**
**PC1**: ${\bar{\text{Bare Soil}}}_{T}$	**2.7376**	**0.8676**	**9.9561**	**.0016**
∆T: ${\bar{\text{Bare Soil}}}_{T}$	**‐718.4483**	**1.7971**	**7.0390**	**.0079**

*Note*: Statistically significant terms (*p* < .05) are marked in bold. For all terms, the degrees of freedom for the Wald *χ* ^2^ test equals 1. Conditional (all variables) and marginal (only fixed terms) pseudo‐*R* ^2^ values are shown at the bottom of the table. Term names correspond to those in Equation 3: ∆T, rate of temperature change; ${\bar{\text{Crop}}}_{T}$, rate of change for cropland land cover; ${\bar{\text{Bare soil}}}_{T}$, rate of change for bare‐soil cover; and PC1, the life‐history axis representing the fast–slow continuum.

*Conditional pseudo‐R* ^*2*^ = 0.25.

*Marginal pseudo‐R* ^*2*^ = 0.067.

Climate warming alone had no significant effect on population trends (Table 1), but the interactive effect of bare‐soil expansion and climate warming did. Species populations subjected to both bare‐soil expansion and climate warming presented negative population trends, whereas species populations in areas of bare‐soil reduction and climate warming presented positive population trends (Figure 2b). In areas where bare‐soil cover was decreasing and temperature declines were observed, populations showed negative trends. Where temperatures decreased and bare soil expanded, populations showed positive population trends (Figure 2b).

### 3.1 Sensitivity analyses

The magnitude of the observed effects, as well as the 95% confidence intervals of the predicted estimates, remained mostly consistent and statistically significant across the datasets with different accuracies of population‐size imputation (Table S4). Conditional pseudo‐*R* ^2^ values (all variables) across these models ranged from 0.18 to 0.25, while marginal pseudo‐*R* ^2^ values (only fixed terms) ranged from 0.069 to 0.074. Models with datasets compiled using a higher *R* ^2^ threshold for data imputation had higher marginal but lower conditional pseudo‐*R* ^2^ values (Table S4). When extreme values of $\bar{\lambda_{T}}$ were removed, models varied in terms of the direction and significance of environmental effects. When 2.5% of the populations with the most negative values of $\bar{\lambda_{T}}$ were removed, model effects showed the same direction but only the interactions between bare‐soil, temperature warming, and the fast–slow continuum remained statistically significant (see Table S2 for further details). We observed a similar trend when populations with both extremely positive and extremely negative values of $\bar{\lambda_{T}}$ were discarded (5% of the data) (Table S5). However, in this case, only the interaction between bare‐soil and temperature warming remained statistically significant (Table S5). When the 2.5% of the populations with the most positive values of $\bar{\lambda_{T}}$ were removed from the analysis, the fast–slow continuum, rate of cropland change and its interaction with the fast–slow continuum were statistically significant (Table S5). Overall, we detected changes in the sensitivity of the models fitted with different datasets and changes in the direction of the effect of temperature warming, although this term was not statistically significant in the original model.

## 4 DISCUSSION

In this study, we showed that terrestrial vertebrate population trends vary according to interactions between land‐cover and temperature changes, as well as with species life histories. Specifically, we found that populations in areas that have experienced recent cropland and bare‐soil expansions are more likely to present negative trends, and that species' population trends are negative in areas that experienced both recent climate warming and bare‐soil expansion. Species with different life‐history strategies showed a distinct response to cropland and bare‐soil expansion, with fast‐lived species tending to show positive population trends, and slow‐lived species tending to display negative trends. Although these findings support the hypothesis that fast‐lived species are better adapted to cope with environmental changes (MacArthur & Wilson, 1967; Pianka, 1970), the main results of our study must be interpreted cautiously. As our sensitivity analysis showed, patterns of population response to climate and land‐use changes are strongly sensitive to the parameters used for the selection of population‐trend data, including the inclusion/exclusion of populations with extreme rates of change.

The effects of human land‐use intensification on biodiversity are well documented and range from habitat fragmentation (e.g. de Oliveira et al., 2017) to community homogenization (e.g. Vellend et al., 2007) and the introduction of invasive species (e.g. Soares et al., 2020). Thus, we expected to observe an effect of land‐cover change on species population trends. We found that land‐cover changes, more precisely those related with changes in the coverage of cropland and bare‐soil, are important determinants of species' population trends, and that species respond differently to these changes according to their position along the fast–slow continuum of life histories. These observations match some of the theoretical predictions from MacArthur and Wilson (1967) and Pianka (1970). Fast‐lived species are better adapted to rapidly increase their populations after environmental perturbations, whereas slow‐lived species are displaced or experience population declines (MacArthur & Wilson, 1967; Pianka, 1970). Our main results can help to explain previous findings that showed that animal communities under land‐use conditions indicative of human disturbance were predominantly occupied by fast‐lived species (e.g. De Palma et al., 2015; Newbold et al., 2013).

It is well established that climate change has affected biodiversity (e.g. Chen et al., 2011; Donnelly et al., 2011; Parmesan & Yohe, 2003), and that its effects are going to become more evident in the future (IPCC, 2021; Newbold, 2018). Previous studies have found a strong association between population declines/extirpations and climate warming in bird, mammal, and reptile populations (Sinervo et al., 2010; Spooner et al., 2018). We did not observe significant effects of climate warming on species population trends, probably because our time window was much shorter than those used in previous studies, which can influence the detectability of climate warming and its effects on species. Unlike land‐use/cover change, which acts as a rapid and easy‐to‐detect driver of environmental change, the effects of climate change are still challenging to detect (e.g. Wu et al., 2015). Furthermore, the fact that climate warming effects are likely to act with a lag further complicates their detectability (e.g. Thompson & Ollason, 2001). Despite this, climate warming had a significant effect when interacting with land‐cover changes. Our main results indicate that these interactions can lead to positive and negative changes in population trends depending on whether both factors follow the same trend (negative), or opposite (positive). This experiment shows that relationships between land‐cover change, climate warming, and population trends are complex, and likely to vary across species.

From a theoretical point of view, we would expect both fast and slow species to maintain average stable populations (MacArthur & Wilson, 1967). However, our main results showed that, on average, fast‐lived species showed positive population trends, while slow species were more likely to present negative population trends (Figure 3b). This can, in part, be explained by the focus of our study. Biodiversity threats, such as habitat destruction, pollution, or the introduction of invasive species, do not act in isolation but in complex clusters, in which multiple drivers can act at the same time and at different scales/intensities (Bowler et al., 2020; Wraith & Pickering, 2018). By focusing only on climate warming and land‐cover changes, we are likely missing other important drivers of biodiversity change that have an impact on species population trends and can be observed in the different average population trends of fast and slow species.

The sensitivity analysis showed that our results were data sensitive. When different *R* ^2^ thresholds (imputation accuracy of population‐size estimates) were used to include or discard populations from the analysis, larger datasets (lower *R* ^2^ thresholds) tended to return models with more statistically significant effects than models with more restricted data (higher *R* ^2^ thresholds). In addition, and independently of *R* ^2^, the imputation methods used to obtain uninterrupted population time‐series could add additional noise, and potentially bias, to the analysis. When populations were discarded based on their rate of population change, the significance, and in one case direction, of the effects varied greatly depending on which populations were discarded from the analysis. We can conclude, therefore, that our results were mostly driven by the effect of a small proportion of populations (2.5%–5% of the total data) with extreme rates of change. These findings suggest that species' sensitivity to environmental change is not uniformly distributed across gradients of environmental change and life‐history strategies but clustered, with some populations and species being particularly sensitive (Leung et al., 2020). These patterns of response were detected only through a sensitivity analysis of our main model, highlighting the sensitivity of time‐series studies to extreme values, and the necessity of including this type of approach in future research.

Overall, the results of our main model showed that populations of species with different life histories respond differently to environmental changes, with populations of fast‐lived species being positively affected by human‐driven land‐cover changes, and populations of slow‐lived species negatively affected. Addtionally, the interactions between climate and land‐cover changes create asymmetric responses on species population rates. Although we observed a clear relationship between life‐history strategies and species response to environmental changes, sensitivity analysis revealed that our results were dependent on the data‐selection process. The addition or exclusion of a small proportion of populations with strong rates of population change can have a large impact on model outcomes. Furthermore, other errors in the data, such as imputation inaccuracies, will add noise and could potentially bias the results. From this, we can conclude that additional and more precise information is needed to fully understand the characteristics that make species sensitive to environmental changes. With land‐use and climate change expected to further increase in the near future (IPCC, 2021; Tilman et al., 2017), it is likely that this asymmetry in the response of fast and slow species to these drivers results in a turnover of community composition. Fast life histories are usually associated with invasive and generalist species (Allen et al., 2017; Cooke et al., 2019), whereas slow life histories are usually associated with specialist species (Cooke et al., 2019). The creation of conditions that favour fast‐lived species, and the decline of slow‐lived species, can lead to a further homogenization of communities, and the loss of important ecosystem services (Clavel et al., 2011). Establishing clear relationships between species traits and sensitivity to environmental change is fundamental to establish effective conservation strategies and halt biodiversity degradation. Here, we have shown that life‐history traits are a useful tool to study species' population responses to climate and land‐cover changes.

## CONFLICT OF INTEREST

All authors declare that they have no conflicts of interest.

## Supporting information


Data S1

Data S2

## Data Availability

The data that support the findings of this study are openly available in Dryad at https://doi.org/10.5061/dryad.djh9w0w3p.
