# Supplementary material for: Species life‐history strategies affect population responses to temperature and land‐cover changes
Source: Glob Chang Biol. 2022 Oct 17;29(1):97–109. doi: 10.1111/gcb.16454 (PMC10092366; doi:10.1111/gcb.16454)
Supplement: Supplementary file 2 — Data S2 [file GCB-29-97-s001.docx]

**Supplementary materials**

**S1. Life-history trait compilation and taxonomic matching**

To describe species life-history strategies we selected five life-history traits: species body mass in grams; species longevity in years; age of sexual maturity in years; number of offspring at each reproductive event; and yearly number of reproductive events. This information was gathered from 24 open-access databases and published scientific articles (Table S1). Since these studies span several decades a taxonomic revision and synonym resolution was needed to make these datasets compatible (Cooke, Bates, & Eigenbrod, 2019; Etard, Morrill, & Newbold, 2020). The names of the species, as given the original publications, were first checked for typographic errors using the R package *taxize* Version 0.9.8 (Chamberlain, Szöcs, Scott Chamberlain, & Eduard Szocs, 2013). Checked or corrected names were then searched in the online repositories of the of the International Union for the Conservation of Nature (IUCN, <http://www.iucnredlist.org> Version 2021-1) and the Integrated Taxonomic Information System (I, <http://www.itis.gov> Version 26-July-2021). Species synonyms, upward taxonomic classification (up to the level of Class) and currently accepted names were retrieved from these online repositories. We select multiple data-sources because to date, there is not a global integrated taxonomic dataset that includes all the information needed (Cooke et al., 2019; Etard et al., 2020). By doing this, we attempted to fill possible gaps and retrieve as much taxonomic information as possible. To create a cohesive taxonomy, we prioritized the IUCN taxonomic information over that of ITIS.

Table S1. Research studies and datasets used to gather life-history trait information. The type of trait contained in each dataset is indicated by an “x”. Life-history traits include; Bm, body-mass (grams); N-offs, number of offspring at each reproductive event (count); Long, longevity (years); Sm, sexual maturity (years); and Rep-Ev, reproductive events (count).

| Dataset | Bm | N-offs | Long | Sm | Rep-Ev |
| --- | --- | --- | --- | --- | --- |
| Amat, 2008 |  | x |  |  | x |
| Capellini, Venditti, & Barton, 2011 | x | x |  |  |  |
| Cassill, 2019 |  | x |  |  |  |
| Gonçalves et al., 2018 | x |  |  |  |  |
| Grimm, Prieto Ramírez, Moulherat, Reynaud, & Henle, 2014 | x | x |  | x |  |
| Jones et al., 2009 | x | x | x | x | x |
| Lobaina, 2014 | x | x |  | x |  |
| Meiri, 2018 | x | X |  |  |  |
| Myhrvold et al., 2015 | x | x | x | x | x |
| Novosolov, Raia, & Meiri, 2013 |  | x |  |  | x |
| Novosolov et al., 2017 | x |  |  |  |  |
| Pacifici et al., 2013 | x |  | x |  |  |
| Clutton-Brock et al., 1999 |  | x |  | x |  |
| Polović, Pešić, Ljubisavljević, & Čadenović, 2013 |  | x |  |  | x |
| Sherman & Jarvis, 2002 |  | x | x |  |  |
| P. W. Sherman, Braude, & Jarvis, 1999 |  | x | x |  |  |
| Schwarz & Meiri, 2017 |  | x |  |  | x |
| Smith et al., 2003 | x |  |  |  |  |
| Stark, Tamar, Itescu, Feldman, & Meiri, 2018 | x | x | x |  |  |
| Trochet et al., 2014 | x | x |  | x |  |
| Scharf et al., 2015 | x | x | x | x | x |
| Verde Arregoitia, Blomberg, & Fisher, 2013 | x |  |  |  |  |
| Wilman et al., 2014 | x |  |  |  |  |
| Bird et al., 2020 |  |  | x | x |  |

**S2. Trait filtering, correction, and aggregation**

The studies included in the life-history trait data compilation encompass a wide variety of research and databases, each with its own objectives and hypothesis. Consequently, traits are presented in different units, with distinct transformation, and labelled with different names. Therefore, it was necessary to implement a series of standards to deal with these differences and allow datasets to be combined. To include a trait into the final dataset: 1) variables had to be clearly described, (i.e., whether estimates refer to adult/juvenile, male/female individuals or minimum/maximum registered values). 2) if any transformation of adjustment has been applied to the original traits, the data and all the information to reverse these transformations must be present either in the original manuscript or into the dataset. 3) the manuscript or dataset must clearly specify if values have been imputed, and the imputation method. If a dataset of research article fails to meet these criteria, we discarded it from the analysis.

Additionally, raw trait data was subjected to a filtering process. First, to detect abnormal values, we produced boxplots for each trait and dataset using species order as the grouping factor. Outliers from these boxplots reflect natural variability of life-history traits or erroneous values. When the number of outliers was low (less than 50), values were individually validated using alternative sources (e.g scientific literature, field guides or expert knowledge). In the cases when the number of outliers was higher than 100, a random sample of 10% was retrieved and checked for errors. If more than 20% of the values from the random sample correspond to erroneous data, the dataset was discarded from the analysis. This process allowed us to deal with some of the errors found in the datasets and add an additional quality control for the trait data. Despite this, it is possible that erroneous estimates of life-history traits were included in our final dataset. Ideally, life-history data would have been checked individually for each species. However, for many species, life-history trait data was only recorded in one dataset, making estimates contrast challenging. In addition, the very large amount of data (multiple traits for more than 20,000 species) made a check of individual trait estimates impractical and unnecessary for the scope of this manuscript.

After the taxonomic resolution, and the pre-processing and checking of life-history trait information, the different datasets were combined. More than 87% of the species were present life-history trait information across multiple datasets. In these cases, trait records were log-transformed, the arithmetic mean was calculated, and then back transformed values were used as species life-history trait estimates for the final dataset. As a result of this final aggregation, we found life-history trait and taxonomic information for 21,126 different species of terrestrial vertebrates (5,313 mammals, 10,442 birds, and 5,371 reptiles). All species trait values were incorporated into a unified dataset that also included species taxonomic information (a full list of synonyms, upward taxonomy to the level of class, and references to the datasets in which each species appears).

**S3. Trait imputation**

The focus of this study was on the terrestrial vertebrate species present in our subsampled LPD. From this sample of species, we missed life-history trait information for nearly 20% of them. To obtain a complete set of life-history traits for all our focus species we performed a random forest phylogenetic trait imputation (Penone et al., 2014). This imputation method can deal with highly dimensional data, and can also model complex interactions and non-linear relationships between species traits (Penone et al., 2014; Stekhoven, Buhlmann, & Bühlmann, 2012). Random forests imputation has been shown to perform better, or similarly, compared with other more time consuming and restricted imputation methods (Penone et al., 2014). Following Penone et al. (2014), we used the first ten eigenvectors, derived from species phylogenetic trees, as our phylogenetic information for the imputation (Diniz-Filho et al., 2012; Diniz Filho, Villalobos, & Bini, 2015). Phylogenetic eigenvectors represent phylogenetic distances among species.

To obtain the phylogenetic eigenvectors, we used the Interactive Tree of Life (iTOL) (<https://itol.embl.de/>) and the TimeTree (Hedges, Marin, Suleski, Paymer, & Kumar, 2015) online interface (<http://www.timetree.org/>) to construct large phylogenetic trees that contained as much of our species as possible. We choose to collect data from different sources because, at the time of the analysis, there was not a unified dataset that contained phylogenetic information for all the species represented in our life-history dataset. Using those data sources, we constructed two consensus species-level phylogenetic trees. The iTOL tree was constructed using the R package *rotl* Version 3.0.10.9000 (Michonneau, Brown, & Winter, 2016), whereas for the TimeTree we used the online interface tool (see [http://www.timetree.org/](https://timetree.org/)). Since the data contained in the iTOL and TimeTree is different, the methods by which phylogenetic trees are build also differ between databases. The iTOL phylogenetic tree is based on a synthetic rooted tree in which all branches have equal lengths (branches do not represent divergence times). Conversely, the phylogenetic tree from TimeTree represent a rooted tree in which branches lengths represent divergence times between species (tree branches have different lengths). Once the phylogenetic trees were constructed, eigenvectors were calculated using a principal coordinate analysis (PCoA) in the R package *PVR* Version 1.0 (Santos, 2018). The first 10 phylogenetic eigenvectors of each species and tree were extracted to be used in the life-history-trait imputations.

Although random forest algorithms are considered Black Box models, some parameters still need to be finely tuned and test before the final imputation. More specifically, the number of trees and interactions as well as the type of phylogenetic information feed to the algorithm can have a great impact on the accuracy of imputations (Penone et al., 2014). To select the adequate configuration of the random forest algorithm, we run a preliminary analysis using a sub sample of 2,501 species from which we had complete trait, taxonomic, and phylogenetic information (form both iTOL and TimeTree). Using this dataset as our template, we randomly removed life-history trait information in different proportions. This way we created four different datasets with different proportions of missing life-history trait data (30%, 50%, 60%, and 80%) along with complete taxonomical and phylogenetic information. Using these datasets, we performed imputations using different numbers of trees, from 50 to 300, for each dataset using different combinations of taxonomic and phylogenetic information:

1. Imputations using only trait information
2. Imputations using trait information + Taxonomic information
3. Imputations using trait information + iTOL eigenvectors
4. Imputations using trait information + TimeTree eigenvectors
5. Imputations using trait information + Taxonomic information + iTOL eigenvectors
6. Imputations using trait information + Taxonomic information + TimeTree eigenvectors

Each of these imputations was repeated 10 times and the overall normalized root-square mean values (NRMSE) were calculated. The NRMSE ranges from 0 to 1 and measures the correspondence between estimated and observed values (Hyndman & Koehler, 2006). Values of NRMSE close to 0 represent higher imputation accuracy while values around 1 are related with low imputation accuracy. In the case of the taxonomic information, and since our random forest algorithm cannot handle factor variables with more than 32 levels (Stekhoven et al., 2012), we transformed the taxonomic information into numerical vectors which were then added into the imputation process. We performed all imputations using the random forest algorithm implemented in the R package *missForest* Version 1.4 (Stekhoven et al., 2012). For the final set of imputation parameters, we also calculated the NRSME for each imputation test and life-history trait (Figure S2).

The results from the preliminary analysis showed that trait completeness, the amount of trait data available for the algorithm, has a strong effect on the accuracy of life-history trait imputation (i.e. lower NRMSE values). As expected, lower proportions of missing data led to greater imputation accuracy in all cases (Figure S1). Results also showed that the incorporation of extra information related to species kinship greatly improved imputation accuracy in all cases (Figure 1). More precisely, imputations that included phylogenetic information present lower NRMSE values than those that only used taxonomic information to account for species kinship (Figure S1). Furthermore, the imputation that used taxonomy and the phylogenetic eigenvectors from iTOL presented a higher imputation accuracy (lower NREMSE values) than the rest of methods (Figure S1). However, NRMSE values between the methods that included kinship information, either in the form of taxonomy or phylogenetic eigenvectors, were small (Figure S1). In terms of number of trees, tests showed that overall, the increase of trees used for the imputation improved accuracy (Figure S1). However, these improvements depend on the proportion of missing data and the type of additional information added to the imputations. For example, when the proportion of missing data was high or only trait information was used, imputations did not improve with the increase of trees. This suggest that the algorithm was not able to accurately impute values when the proportion of real information was low, or kinship wasn’t considered (Figure S1).


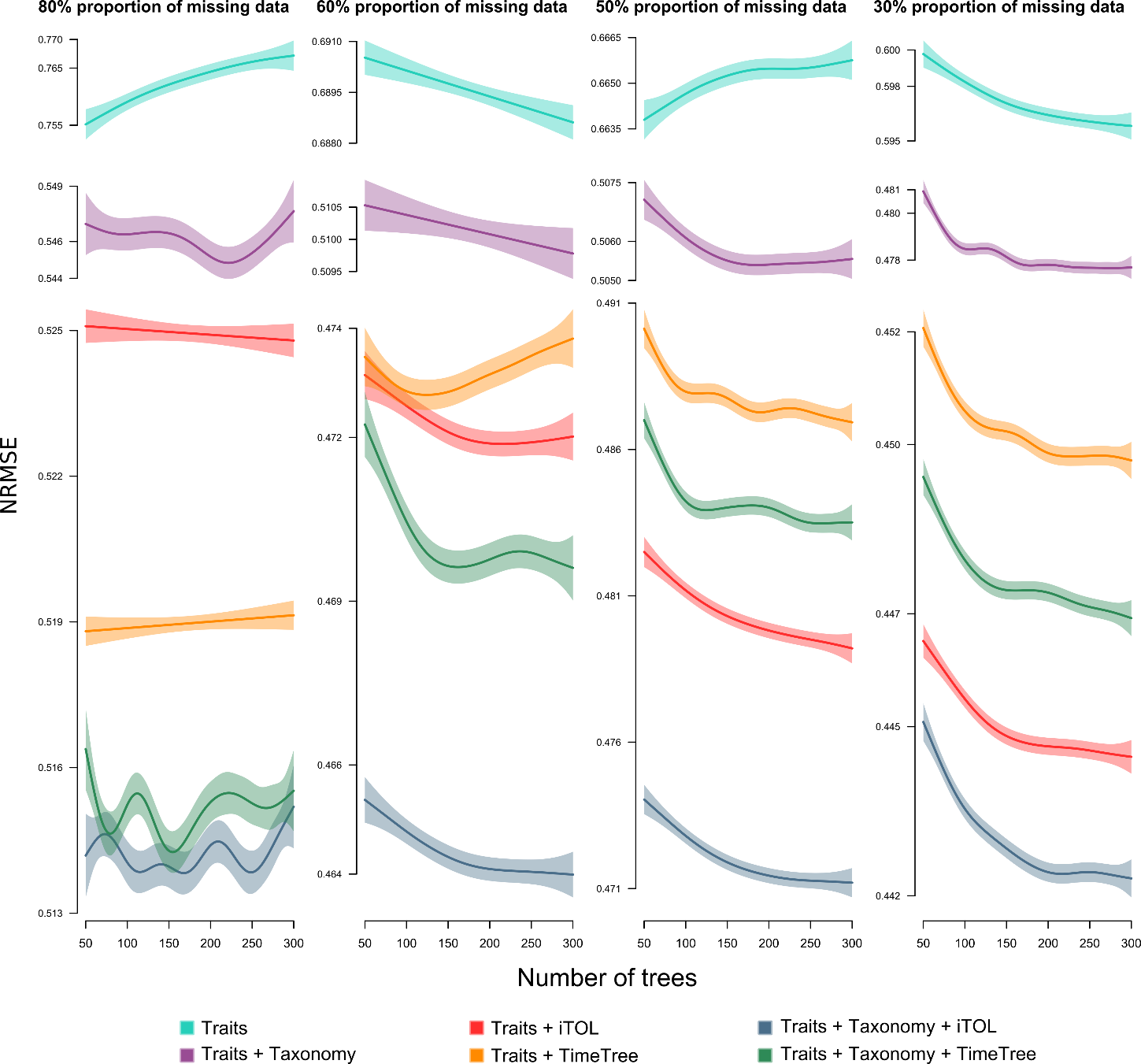
Figure S1. Mean real imputation errors (Normalized Root Mean Square Errors; **NRMSE**) for random forests imputations using different proportions of missing trait information and numbers of trees. The different coloured lines represent different combinations of trait data and supplementary information; **clear blue**, only trait information; **purple,** traits and taxonomic information; **red,** traits and the first 10 eigenvectors of iTOL; **gold,** traits and the first 10 TimeTree-derived eigenvectors; **dark blue,** traits and the first 10 iTOL-derived eigenvectors along with taxonomic information; and **green,** traits and the first 10 TimeTree-derived eigenvectors plus species’ taxonomic information. Solid lines represent the fitted values of generalized additive models (GAM) with smoothed partial residuals regression splines, calculated by generalized maximum likelihood, fitted using NRMSE values of each imputation/test. GAMs were adjusted using the R package *gam* Version 1.20 (Hastie, 2020). The different percentages of missing data were obtained by removing, at random, trait values from a dataset of 2,501 species for which we had complete trait, phylogenetic, and taxonomic information.

In summary: 1) phylogenetic information from iTOL produced greater imputation accuracy than the phylogenetic data from TimeTree; 2) in order to keep precise imputations, the percentage of missing data had to be low; 3) When missing information was between 30% and 60% imputation accuracy increased with the number of trees; 4) in all cases, the combined inclusion of phylogenetic and taxonomic information improved imputation accuracy, lower values of NRMSE (Figure S2).

Overall, the combination of high trait completeness and kinship information were the approaches that showed the greatest imputation accuracy and therefore this was the approach used to perform the final imputation (Figure S2). Based on the results from the test we controlled the proportion of missing information by selecting species with higher proportions of trait completeness for the final imputation. Following this approach, a total of 9,618 species (2,765 mammals, 4,638 birds and 2,215 reptiles) species were included into the final imputation. This selection included our focus species, those presented in our sampled LPD, as well as other species. This way we achieve two objectives. First, we increased the amount of information available for the random forest algorithm (the proportion of missing data of this dataset was below 30%); and second, we increase the number of species present for the life-history classification (Figure S2-3). We set the number of trees and maximum number of iterations of the random forest imputation to 150 and 15 based on the results from the preliminary tests.

In addition to life-history trait information, we added the 10 first eigenvectors derived from TimeTree. Although iTOL showed slightly higher levels of imputation accuracy during the tests (Figure S1), TimeTree had a better coverage of the species selected (more than 68%) and, since the differences in accuracy were low between the two phylogenetic datasets (both present values of NRMSE below 0.45, Figure S2), we considered that including more information into the imputation overcomes the possible loss of accuracy. Finally, and since we did not have phylogenetic information for all species, we added the upward taxonomic information of all species as additional kinship information. The final imputed life-history trait values were calculated as the arithmetic mean of 100 imputations and added to the final life-history trait dataset for the calculation of species life-history strategies (Figure S3).


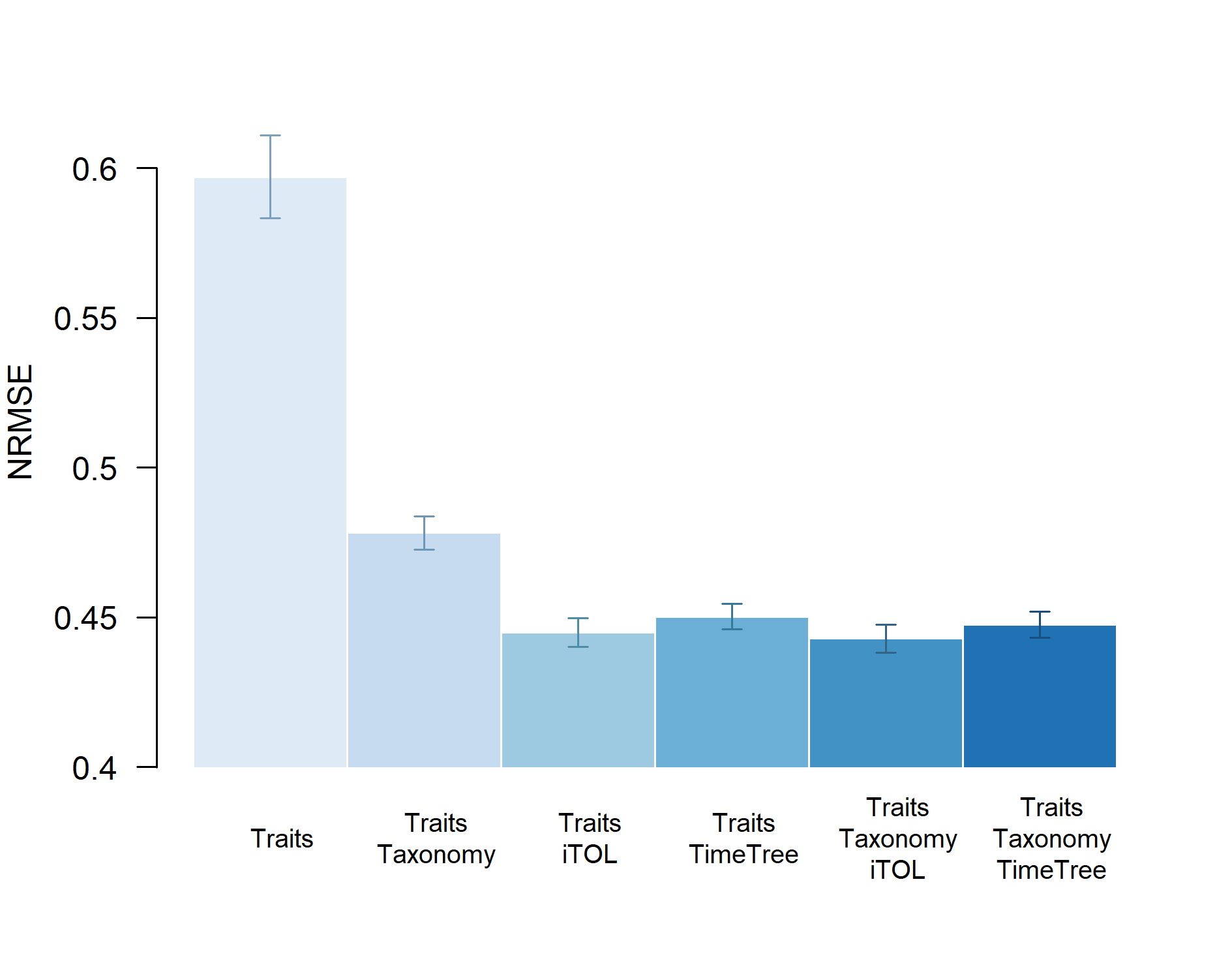


Figure S2. Normalized root-square mean values (NRMSE) for each dataset combination. Displayed NRMSE are based on imputations using 150 trees and 15 maximum iterations of the random-forest algorithm. Lower NRMSE values are related with higher imputation accuracy while NRMSE values closer to 1 are associated with low imputation accuracy. Bars represent approximate confidence intervals of the imputations (2 times the standard deviation).


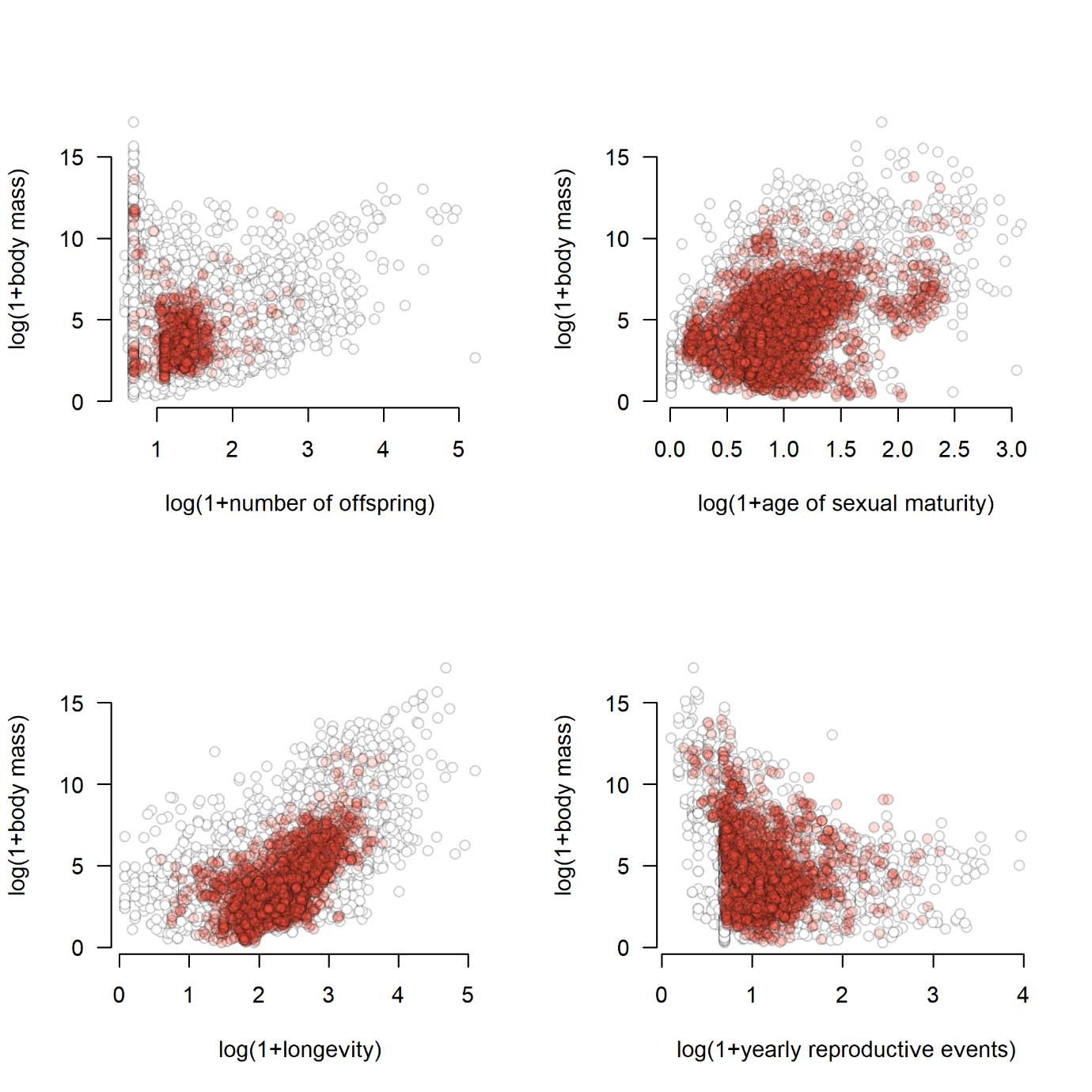


Figure S3. Plots of the pairwise relationships between log-transformed values of the life-history traits. Hollow circles represent pairs of non-imputed life-history-trait values, whereas red-filled circles represent pairs of life-history traits where at least one of the two values was imputed using a random forest phylogenetic imputation (details see text S.3).

**S4. Species life-history strategies (the fast-slow continuum)**

To characterize our species life-history strategies we used the fast-slow continuum classification scheme (Healy, Ezard, Jones, Salguero-Gómez, & Buckley, 2019; Read & Harvey, 1989; Stearns, 1983). To classify our species along the fast-slow continuum, first we need to adjust our life-history traits according to species body-mass and phylogenetic relationship (e.g Dobson & Oli, 2007). This is because, body size is an important factor that impacts other important life-history traits such as longevity (Stearns, 1983). For example, large bodied species are likely to spend more time growing, are more likely to present late sexual maturity, and have greater life-spans and fewer offspring than small, fast growing species. In turn, this makes the comparison of life-history between species of different size challenging (Dobson & Oli, 2007). Furthermore, phylogeny is an important factor shaping both species body-size and life-histories. Closely related species are more likely to share similar life-history traits and body size than non-related species. Since we are interested in comparing the life-history strategies of species across three different taxonomic classes, the effects of species kinship also need to be controlled. To control the effects of body-mass on our life-history traits, we regress each life-history trait against body-mass. For this we used linear mixed-effects models (LMMs), fitted using restricted maximum likelihood. Since we wanted to control for the effects of kinship, but we lack complete phylogenetic information for our species (see section S3), we added the upward taxonomic classification, up to the level of class, as a random effect on the LMM. Taxonomic classification was added following a nested structure (family nested within Order nested within Class) to allow the different groups of species to have distinct intercepts in the model. To control for the skewness of the trait data (see Figure S3), all variables were log-transformed before the analysis. To control for residual heteroscedasticity, we added an exponential variance structure. This structure modelled the variance of the residuals as σ^2^ multiplied by an exponential function of the covariance matrix of our explanatory variables multiplied by a parameter δ, which allows for a different spread of residuals across factor levels (Zuur, Ieno, Walker, Saveliev, & Smith, 2009). The residuals from these models were used as life-history trait values adjusted for body mass and species kinship (“adjusted traits” from now on). More details about these models can be observed in Table S3. LMMs were fitted using the lme function of the R package *nlme* Version 3.1-157 (Pinheiro, Bates, DebRoy, & R Core Team, 2021).

After the correction, the adjusted traits were subjected to a Principal Component Analysis (PCA). The three first axis of this analysis encompass more than 80% of the total life-history trait variation and represents different aspects of the species life-histories (Healy et al., 2019) (Table S4). The first axis of the PCA showed the trade-offs expected from the fast-slow continuum, this is a negative relationship between fecundity and ageing. This axis explained 32% of the total life-history trait variance. Therefore, we considered this axis to represent the fast-slow continuum. In the case of the second PC axis, there were no trade-offs between variables (all loaded positively on this axis, Table S4 and Figure S4). However, this axis represented mostly yearly reproductive events, with a contribution of nearly 70% of the total variance of this axis (Table S4 and Figure S4). As for the third axis, the variables with the greater contribution were number of offspring and longevity, which also showed negative loading values (Table S4 and Figure S4).

Table S3. Linear mixed-effects models used to adjust trait estimates to account for the effects of body mass and taxonomic relationships of mammals, birds and reptiles in our analysis. Fixed-effects coefficients and random effects are presented. Fixed effects contain the parameter estimate for each explanatory variable value (**value**), their standard deviation (**stDev**), and the **t-value** (derived using the Satterthwaite t-test approximation, see Kuznetsova et al., 2017) along with its corresponding P value (**P**). We present the variance and standard deviation associated with each random effect (**Variance** and **stDev** respectively), the fraction of total variance explained by those factors (**Exp-var**), the total variance explained by the random effects (**Total var**), and the residual variance unexplained by the random effects (**Residual**). Models have a sample size of 9,618 species (4,638 birds, 2,765 mammals, and 2,215 reptiles). Random effects included were Family (**Fam**) nested within Order (**Or**) nested within Class (**Cl**).

|  | **Longevity** | | | | **Sexual Maturity** | | | |
| --- | --- | --- | --- | --- | --- | --- | --- | --- |
|  | **value** | **stDev** | **t** | **P** | **value** | **stDev** | **t** | **P** |
| **intercept** | 1.802 | 0.23150 | 7.782 | 0.000 | 0.805 | 0.266 | 3.029 | 0.003 |
| **log(Bm)** | 0.147 | 0.004 | 38.020 | 0.000 | 0.071 | 0.002 | 33.355 | 0.000 |
|  | **Random** | | | | **Random** | | | |
|  | **Variance** | **stDev** | **Exp-var** | **Total var** | **Variance** | **stDev** | **Exp-var** | **Total var** |
| **Cl** | 0.146 | 0.383 | 0.364 | 0.742 | 0.204 | 0.452 | 0.614 | 0.785 |
| **Cl/Or** | 0.108 | 0.328 | 0.268 |  | 0.057 | 0.239 | 0.171 |  |
| **Cl/Or/Fam** | 0.044 | 0.211 | 0.110 |  | 0.043 | 0.207 | 0.129 |  |
| **Residual** | 0.104 | 0.322 |  |  | 0.028 | 0.169 |  |  |
|  | **Number of offspring** | | | | **Yearly reproductive events** | | | |
|  | **value** | **stDev** | **t** | **P** | **value** | **stDev** | **t** | **P** |
| **intercept** | 1.255 | 0.450 | 2.788 | 0.005 | 1.098 | 0.088 | 2.788 | 0.005 |
| **log(Bm)** | 0.056 | 0.003 | 17.364 | 0.000 | -0.027 | 0.003 | 17.364 | 0.000 |
|  | **Random** | | | | **Random** | | | |
|  | **Variance** | **stDev** | **Exp-var** | **Total var** | **Variance** | **stDev** | **Exp-var** | **Total var** |
| **Cl** | 0.594 | 0.770 | 0.685 | 0.794 | 0.020 | 0.141 | 0.109 | 0.195 |
| **Cl/Or** | 0.094 | 0.307 | 0.109 |  | 0.016 | 0.126 | 0.087 |  |
| **Cl/Or/Fam** | 0.122 | 0.350 | 0.141 |  | 0.047 | 0.218 | 0.260 |  |
| **Residual** | 0.056 | 0.237 |  |  | 0.099 | 0.315 |  |  |

Table S4. Factor loadings and contribution percentage to the principal components of each life-history trait variable (number of offspring, longevity, yearly reproduction events, and sexual maturity). Correlation of the different principal components (**PC**) with body mass (**Correlation Bm**) are also shown here along with the eigenvectors, eigenvalues, explained variance and cumulative explained variance (**Exp-Variance** and **Cum-Variance** respectively) for the three main life-history axes.

|  | ***PC1*** | | ***PC2*** | | ***PC3*** | |
| --- | --- | --- | --- | --- | --- | --- |
|  | **Loadings** | **Contribution** | **Loadings** | **Contribution** | **Loadings** | **Contribution** |
| **Number of offspring** | 0.5921 | 35.0619 | 0.1281 | 1.6424 | -0.5841 | 34.1286 |
| **Longevity** | -0.4794 | 22.3968 | 0.0332 | 0.1107 | -0.8034 | 64.5570 |
| **Yearly reproduction events** | 0.2963 | 8.7843 | 0.8351 | 69.7457 | 0.0587 | 0.3447 |
| **Sexual maturity** | -0.5759 | 33.1668 | 0.5338 | 28.5011 | 0.0984 | 0.9694 |
| **Correlation Bm** | -0.009 | | 0.01 | | 0.007 | |
| **Eigenvalues** | 1.2963 | | 1.0549 | | 0.9524 | |
| **Exp-Variance** | 32.4093 | | 26.3726 | | 17.4064 | |
| **Cum-Variance** | 32.4093 | | 58.7821 | | 82.5935 | |


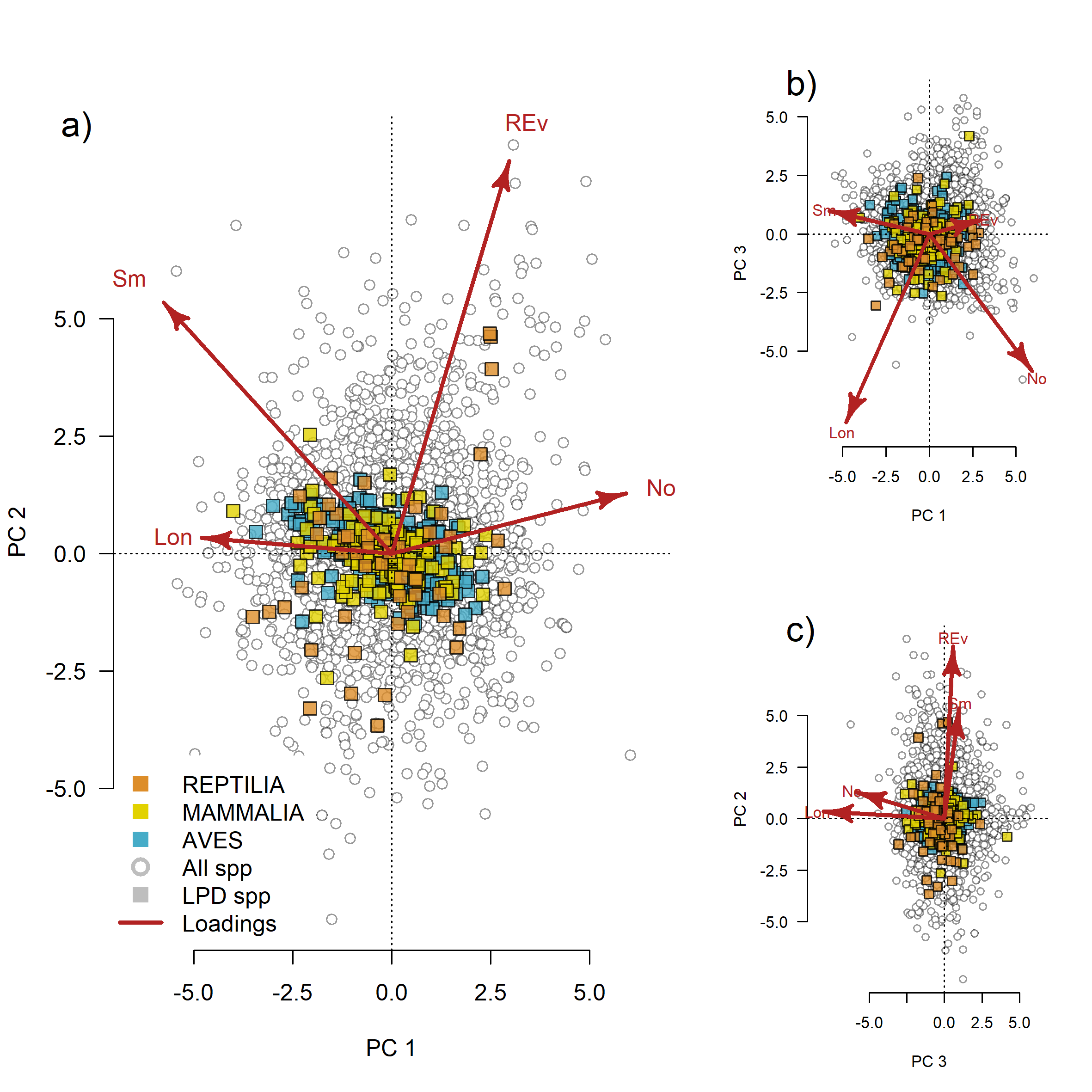


Figure S4. Relationships of the score values for the three main axis of the Principal Component Analysis (PCA); **a)** PC1 and PC2; **b)** PC1 and PC3; and **c)** PC3 and PC2. Each point represents the position of a species in the life-history diversity space according to their combination of life-history traits. The different colours represent the main vertebrate classes included in the sampled LPD dataset (471 different species; 278 birds; 142 mammals, and 51 reptiles). The grey circles represent the rest of the species used for the life-history classification (9,618 species; 4,638 birds; 2,765 mammals; and 2,215 reptiles). Arrows represent the direction and influence of the loading values for each life-history variable (**Sm**, sexual maturity; **No**, number of offspring; **Lon**, longevity; and **REv**, yearly reproductive events) on the different life-history axes. Loading values have been 10x rescaled for representation purposes.

**S5. Sensitivity analysis methods**

The set of populations included in the analysis, their spatial location, and their time-series length can have a strong impact on the capacity to detect environmental and population changes (Wauchope et al., 2019). The data used to run the main analysis contains populations for which population estimates had been imputed using GAMs or log-linear regression (details see main text **Species population trends** section for more details). Populations with imputed values are associated with an *R*^2^ value (derived from the model used to impute the data) that serves as a measure of imputation quality: higher values of *R*^2^ are associated with high imputation accuracy (high quality) and lower values are associated with low imputation accuracy (low quality). To test the robustness of the observed results, we re-ran the final selected linear mixed-effects model (LMM) (see **equation 4** in main text) using three different datasets containing distinct subsets of populations from the dataset used to run the main model. Populations were selected by modifying the *R*^2^ threshold of populations with imputed data from 0.5 (used for the main analysis) to 0.3, 0.7, and 0.9. This filtering resulted in three datasets; *R*^2^-0.3 = 1,108 populations; *R*^2^-0.7 = 996 populations; and *R*^2^-0.9 = 915 populations.

Additionally, to test the impacts of population selection on the results observed, we re-ran the main model on 3 different subsets of our dataset, specifically subsets in which populations with extreme rates of population change ($\bar{\lambda_{T}}$) were selectively removed (e.g Leung et al., 2020; Murali, de Oliveira Caetano, Barki, Meiri, & Roll, 2022). This way a **lower-extreme** dataset was created by selecting all the populations that presented a value of $\bar{\lambda_{T}}$ above the 2.5^th^ percentile (the most negative declines were removed). Similarly, an **upper-extreme** dataset was created by selecting all the populations with values below the 97.5^th^ percentile of $\bar{\lambda_{T}}$ (the most positive increases were removed). A third dataset, **both-extremes**, was created by selecting the populations between the 2.5-97.5^th^ percentiles of $\bar{\lambda_{T}}$ (both extremely negative and positive rates of population change were removed). The final sample size was 1,018 populations for **both-extremes,** and 1,045 populations for the **lower/upper-extreme** datasets respectively.

All models were fitted using restricted maximum likelihood, implemented in the R package *lme4* Version 1.1-26 (Bates, Mächler, Bolker, & Walker, 2015). Random sampling was performed using the R *seet.seed* and *sample* functions, both implemented in the R package *base* Version 4.0.2 (R Core Team, 2021). Model fit was evaluated using conditional and marginal pseudo-*R^2^* values calculated by the R package *MuMin* Version 1.43.17 (Barton, 2020). Wald Chi-square test was implemented using the R package *car* Version 3.0-9 (Fox & Weisberg, 2019).

**S6. Sensitivity analysis results**

Overall, fixed effects were broadly consistent across datasets irrespective of different thresholds of *R^2^* to subset the data. In terms of significance of effects, rate of cropland change was the only that differed depending on *R^2^* threshold used. This term was statistically significant when populations with *R^2^* values of 0.3-0.5 were included in the analysis, but non-significant when a more restrictive threshold for the inclusion of populations was used (*R^2^* values between 0.7 and 0.9). Despite this, the direction of all the effects remained constant across the different datasets (Table S5). These results suggest that rate of cropland change is sensitive to changes in data quality and sample size, whereas the response of the rest of the terms remained constant across datasets (Table S5).

The results from the models in which extremely low/high values of $\bar{\lambda_{T}}$ were removed showed greater differences from the main model. None of these models match the results obtained in the main model in terms of the statistical significance of its effects. When the upper and lower-most $\bar{\lambda_{T}}$ were removed from the data (**both extremes**), only the interaction between climate warming and bare-soil land-cover remained statistically significant (Table S6). For the same dataset (**both extremes)**, climate warming also showed a change in its effect direction when compared to the main model (Table S6). When the populations with the lowest $\bar{\lambda_{T}}$ were removed from the analysis (**lower-extreme**), the effects of the interaction of the fast-slow continuum and bare-soil land-cover, and the interaction of climate warming and bare-soil land-cover matched the main model in both statistical significance and effects direction (Table S6). In this case, all effects directions matched the pattern observed in the main model (Table S6). For the model in which the populations with the higher $\bar{\lambda_{T}}$ were removed (**upper-extreme**), the fast-slow continuum, rate of cropland change, and the interaction between these variables showed the same effects direction and statistical significance as the main model (Table S6). The effects direction of the rest of the terms matched the same pattern as the main model, with the exception of climate warming (Table S6).

|  | **0.3; N= 1,108** | | | **0.5; N= 1,072** | | | **0.7; N= 996** | | | **0.9; N= 915** | | |
| --- | --- | --- | --- | --- | --- | --- | --- | --- | --- | --- | --- | --- |
|  | Estimate | *χ^2^* | *p* | Estimate | *χ^2^* | *p* | Estimate | *χ^2^* | *p* | Estimate | *χ^2^* | *p* |
| (Intercept) | 0.0022 ± 0.0028 | 0.6097 | 0.4349 | 0.0025 ± 0.0032 | 0.8711 | 0.3507 | 0.0036 ± 0.0029 | 1.5509 | 0.2130 | **0.0055 ± 0.0029** | **3.6136** | **0.0573 .** |
| $\Delta T$ | 0.003 ± 0.0024 | 0.0137 | 0.9068 | 0.0246 ± 3.3658 | 0.0045 | 0.9463 | <0.0001 ± 0.0025 | 0.0000 | 0.9970 | 0.0003 ± 0.0026 | 0.0166 | 0.8976 |
| PC1 | **0.0052 ± 0.0023** | **5.3038** | **0.0213*** | **0.0052 ± 0.0023** | **5.0484** | **0.0246*** | **0.0059 ± 0.0024** | **5.9357** | **0.0148*** | **0.0065 ± 0.0024** | **7.3435** | **0.0067**** |
| $\bar{Crop}_{T}$ | **-1.4983 ± 0.6796** | **4.8599** | **0.0275*** | **-1.4970 ± 0.6943** | **4.6512** | **0.0310*** | -1.2895 ± 0.7159 | 3.2443 | 0.0717 . | -1.1153 ± 0.7135 | 2.4430 | 0.1181 |
| $\bar{Bare Soil}_{T}$ | 1.4428 ± 1.7695 | 0.6649 | 0.4148 | 5.1350 ± 3.015 | 0.6105 | 0.4346 | 2.3248 ± 1.9109 | 1.4802 | 0.2237 | 2.3564 ± 1.8935 | 1.5487 | 0.2133 |
| PC1: $\bar{Crop}_{T}$ | **1.8382 ± 0.5713** | **10.3509** | **0.0013**** | **1.8960 ± 0.6018** | **9.9276** | **0.0016**** | **1.7831 ± 0.6369** | **7.8372** | **0.0051**** | **1.7055 ± 0.6373** | **7.1612** | **0.0074**** |
| PC1: $\bar{Bare Soil}_{T}$ | **2.745 ± 0.8555** | **10.2942** | **0.0013**** | **2.7380 ± 0.8676** | **9.9562** | **0.0016**** | **2.8866 ± 0.8921** | **10.4687** | **0.0012**** | **2.9393 ± 0.8982** | **10.7091** | **0.0011**** |
| $\Delta T$ : $\bar{Bare Soil}_{T}$ | **-4.773 ± 1.7726** | **7.2516** | **0.0071**** | **-718.4 ± 270.8** | **7.0390** | **0.0080**** | **-5.8548 ± 1.9913** | **8.6440** | **0.0033**** | **-6.1629 ± 2.0646** | **8.9100** | **0.0028**** |
| **C-pseudo-*R*^2^** | 0.2557 | | | 0.2488 | | | 0.2343 | | | 0.1869 | | |
| **M-pseudo-*R*^2^** | 0.0685 | | | 0.0668 | | | 0.0687 | | | 0.0736 | | |

Table S5. Estimate (± standard deviation) and results of an ANOVA type III Wald Chi-square test for species population trends models using datasets sampled at different *R*^2^ thresholds. Dataset size for each model is denoted by **N** and presented along with the *R^2^* threshold used for the filtering of populations at the top of each set of metrics. Statistically significant terms are marked in bold with stars indicating the level of significance (P < 0.1 = .; P < 0.05 = *; P < 0.01 = **; and P < 0.001 = ***). The degrees of freedom for all terms were equal to 1. Model conditional and pseudo-R^2^ values are shown at the bottom of the table. Fixed effects included in the models were: $\Delta T$, rate of warming; $\bar{Crop}_{T}$, rate of change for cropland land-cover; $\bar{Bare soil}_{T}$, rate of change for bare soil cover; and PC1, the fast-slow continuum (see also equation 4 in the main text). Green shaded fields denote agreement between the sensitivity test models and the main model.

Table S6. Estimate (± standard deviation) and results of an ANOVA type III Wald Chi-square test for the species population trends models using datasets from which populations with extreme populations trends have been removed. In the case of the **both-extremes** model, populations with extremely negative and positive population trends were removed (data trimmed at the 2.5 and 97.5 percentiles). For the **lower-extreme** models, populations with populations trends below the 2.5^th^ percentile were discarded. In the case of **upper**-**extreme** values, population that showed population trends above the 97.5 percentile were discarded. Dataset size for each model is denoted by **N** and presented along with the model name. Statistically significant terms are marked in bold with stars indicating the level of significance (P < 0.1 = .; P < 0.05 = *; P < 0.01 = **; and P < 0.001 = ***). Degrees of freedom for all terms were equal to 1. Model conditional and pseudo-R^2^ values are shown at the bottom of the table. Fixed effects included in the models were: $\Delta T$, rate of warming; $\bar{Crop}_{T}$, rate of change for cropland land-cover; $\bar{Bare soil}_{T}$, rate of change for bare soil cover; and PC1, the fast-slow continuum (see also equation 4 in the main text). Green shaded fields denote agreement between the sensitivity test models and the main model.

|  | **Both-extremes; N= 1,018** | | | **Lower-extremes; N= 1,045** | | | **Upper-extremes; N= 1,045** | | |
| --- | --- | --- | --- | --- | --- | --- | --- | --- | --- |
|  | Estimate | χ^2^ | *p-value* | Estimate | χ^2^ | *p-value* | Estimate | χ^2^ | *p-value* |
| (Intercept) | 0.0033 ± 0.0025 | 1.7494 | 0.1860 | **0.0068 ± 0.0029** | **5.4445** | **0.0196*** | -0.0012 ± 0.003 | 0.1503 | 0.6983 |
| $\Delta T$ | -0.0497 ± 0.2709 | 0.0337 | 0.8543 | 0.0891 ± 0.3176 | 0.0787 | 0.7791 | -0.1347 ± 0.3287 | 0.1681 | 0.6818 |
| PC1 | 0.0018 ± 0.0017 | 1.0309 | 0.3099 | 0.0028 ± 0.0020 | 1.9454 | 0.1631 | **0.0039 ± 0.0021** | **3.5214** | **0.0606 .** |
| $\bar{Crop}_{T}$ | -0.8173 ± 0.5484 | 2.2209 | 0.1362 | -0.4713 ± 0.6377 | 0.5462 | 0.4599 | **-1.9383 ± 0.6402** | **9.1673** | **0.0025**** |
| $\bar{Bare Soil}_{T}$ | 2.7716 ± 2.2939 | 1.4599 | 0.2270 | 5.4665 ± 2.6299 | 4.3205 | 0.0377* | 2.4317 ± 2.7658 | 0.7730 | 0.3793 |
| PC1: $\bar{Crop}_{T}$ | 0.5483 ± 0.51 | 1.1557 | 0.2824 | 0.3243 ± 0.5863 | 0.3059 | 0.5802 | **2.0806 ± 0.5396** | **14.8656** | **0.0001***** |
| PC1: $\bar{Bare Soil}_{T}$ | 0.4963 ± 0.6851 | 0.5249 | 0.4688 | **2.0439 ± 0.7567** | **7.2968** | **0.0069**** | 1.0751 ± 0.8117 | 1.7544 | 0.1853 |
| $\Delta T$ : $\bar{Bare Soil}_{T}$ | **-396.7622 ± 212.47** | **3.4869** | **0.0619 .** | **-778.9622 ± 240.23** | **10.5142** | **0.0012**** | -338.9939 ± 257.67 | 1.7307 | 0.1883 |
| **C-pseudo-*R*^2^** | 0.2038 | | | 0.2575 | | | 0.2405 | | |
| **M-pseudo-*R*^2^** | 0.0223 | | | 0.073 | | | 0.0353 | | |

Table S7. Cross walking table for the transformation of ESA CCI land-use discrete data into unified land-cover data (adapted from Li et al., 2017). Unified land covers are: TBe, tree broadleaf evergreen; TBd, tree broadleaf deciduous; TNe, tree needleleaf evergreen; TNd, tree needleleaf deciduous; SBe, shrub broadleaf evergreen; SBd, shrub broadleaf deciduous; SNe, shrub needleleaf evergreen; SNd, shrub needleleaf deciduous; NB, natural grass; Cr, crop; Bs, bare-soil; Wr, water; S/I, snow/ice; Ur, urban; and Nt, no data. This table provided the information to transform the pixel values (Pix) from the ESA CCI LCP (<http://www.esa-landcover-cci.org>) into percentage of land-cover for the aforementioned land-cover types.

|  |  | **Unified land-cover** | | | | | | | | | | | | | | |
| --- | --- | --- | --- | --- | --- | --- | --- | --- | --- | --- | --- | --- | --- | --- | --- | --- |
| **Pix** | **ESA CCI description** | **TBe** | **TBd** | **TNe** | **TNd** | **SBe** | **SBd** | **SNe** | **SNe** | **NG** | **Cr** | **Bs** | **Wr** | **S/I** | **Ur** | **Nt** |
| 0 | No data |  |  |  |  |  |  |  |  |  |  |  |  |  |  | 100 |
| 10 | Cropland, rainfed |  |  |  |  |  |  |  |  | 10 | 90 |  |  |  |  |  |
| 11 | Herbaceous cover |  |  |  |  |  |  |  |  | 10 | 90 |  |  |  |  |  |
| 12 | Tree or shrub cover |  |  |  |  |  | 70 |  |  |  | 30 |  |  |  |  |  |
| 20 | Cropland, irrigated or post-flooding |  |  |  |  |  |  |  |  |  | 100 |  |  |  |  |  |
| 30 | Mosaic cropland (>50%) / natural vegetation (tree, shrub, herbaceous cover)(<50%) | 5 | 5 |  |  | 5 | 5 | 5 |  | 15 | 60 |  |  |  |  |  |
| 40 | Mosaic natural vegetation (tree, shrub, herbaceous cover (>50%)/ cropland (<50%) | 7.5 | 7.5 |  |  | 10 | 15 | 10 |  | 30 | 20 |  |  |  |  |  |
| 50 | Tree cover, broadleaved, evergreen, close to open (>15%) | 90 |  |  |  | 5 | 5 |  |  |  |  |  |  |  |  |  |
| 60 | Tree cover, broadleaved, deciduous, closed to open (>15%) |  | 50 |  |  |  | 20 |  |  | 30 |  |  |  |  |  |  |
| 61 | Tree cover, broadleaved, deciduous, croloasdeldea (f> 40%) |  | 70 |  |  |  | 15 |  |  | 15 |  |  |  |  |  |  |
| 62 | Tree cover, broadleaved, deciduous, open (15-40%) |  | 30 |  |  |  | 25 |  |  | 45 |  |  |  |  |  |  |
| 70 | Tree cover, needleleaved, evergreen, closed to open (>15%) |  |  | 70 |  | 5 | 5 | 5 |  | 15 |  |  |  |  |  |  |
| 71 | Tree cover, needleleaved, evergreen, closed (>40%) |  |  | 70 |  | 5 | 5 | 5 |  | 15 |  |  |  |  |  |  |
| 72 | Tree cover, needleleaved, evergreen, open (15-40%) |  |  |  |  |  |  | 25 |  | 45 |  |  |  |  |  |  |
| 80 | Tree cover, needleleaved, deciduous, closed to open (>15%) |  |  |  | 50 | 2.5 | 2.5 | 2.5 | 12.5 | 30 |  |  |  |  |  |  |
| 81 | Tree cover, needleleaved, deciduous, closed (>40%) |  |  |  | 70 | 5 | 5 | 5 |  | 15 |  |  |  |  |  |  |
| 82 | Tree cover, needleleaved, deciduous, open (15-40%) |  |  |  | 30 |  |  |  | 25 | 45 |  |  |  |  |  |  |
| 90 | Tree cover, mixed leaf type (broadleaved and needleleaved) |  | 30 | 20 | 10 | 5 | 5 | 5 |  | 25 |  |  |  |  |  |  |
| 100 | Mosaic tree and shrub (>50%) / herbaceous cover (<50%) | 10 | 20 | 5 | 5 | 5 | 10 | 5 |  | 40 |  |  |  |  |  |  |
| 110 | Mosaic herbaceous cover (>50%) / tree and shrub (<50%) | 5 | 10 | 5 |  | 5 | 10 | 5 |  | 60 |  |  |  |  |  |  |
| 120 | Shrubland |  |  |  |  | 15 | 30 | 15 |  | 40 |  |  |  |  |  |  |
| 121 | Shrubland evergreen |  |  |  |  | 30 |  | 30 |  | 40 |  |  |  |  |  |  |
| 122 | Shrubland deciduous |  |  |  |  |  | 60 |  |  | 40 |  |  |  |  |  |  |
| 130 | Grassland |  |  |  |  |  |  |  |  | 100 |  |  |  |  |  |  |
| 140 | Lichens and mosses |  |  |  |  |  |  |  |  | 100 |  |  |  |  |  |  |
| 150 | Sparse vegetation (tree, shrub, herbaceous cover) (<15%) |  | 5 | 5 |  |  | 5 | 5 |  | 30 |  | 50 |  |  |  |  |
| 152 | Sparse shrub (<15%) |  |  |  |  |  | 10 | 10 |  | 30 |  | 50 |  |  |  |  |
| 153 | Sparse herbaceous cover (<15%) |  |  |  |  |  |  |  |  | 50 |  | 50 |  |  |  |  |
| 160 | Tree cover, flooded, fresh or brackish water | 37.5 | 37.5 |  |  |  |  |  |  | 25 |  |  |  |  |  |  |
| 170 | Tree cover, flooded, saline water | 75 |  |  |  | 25 |  |  |  |  |  |  |  |  |  |  |
| 180 | Shrub or herbaceous cover, flooded, fresh/saline/brackish water |  |  |  |  |  | 25 | 15 |  | 60 |  |  |  |  |  |  |
| 190 | Urban areas |  |  |  |  |  |  |  |  |  |  |  |  |  | 100 |  |
| 200 | Bare areas |  |  |  |  |  |  |  |  |  |  | 100 |  |  |  |  |
| 201 | Consolidated bare areas |  |  |  |  |  |  |  |  |  |  | 100 |  |  |  |  |
| 202 | Unconsolidated bare areas |  |  |  |  |  |  |  |  |  |  | 100 |  |  |  |  |
| 210 | Water bodies |  |  |  |  |  |  |  |  |  |  |  | 100 |  |  |  |
| 220 | Permanent snow and ice |  |  |  |  |  |  |  |  |  |  |  |  | 100 |  |  |

**Bibliography**

Amat, F. (2008). Exploring female reproductive tactics: Trade-offs between clutch size, egg mass and newborn size in lacertid lizards. *Herpetological Journal*, *18*(3), 147–153.

Barton, K. (2020). MuMin: Multi-Model Inference. *R Package Version 1.43.17*. Retrieved from https://cran.r-project.org/package=MuMIn

Bates, D., Mächler, M., Bolker, B., & Walker, S. (2015). Fitting Linear Mixed-Effects Models Using lme4. *Journal of Statistical Software*, *67*(1). https://doi.org/10.18637/jss.v067.i01

Bird, J. P., Martin, R., Akçakaya, H. R., Gilroy, J., Burfield, I. J., Garnett, S. T., … Butchart, S. H. M. (2020). Generation lengths of the world’s birds and their implications for extinction risk. *Conservation Biology*, *34*(5), 1252–1261. https://doi.org/10.1111/cobi.13486

Capellini, I., Venditti, C., & Barton, R. A. (2011). Placentation and Maternal Investment in Mammals. *The American Naturalist*, *177*(1), 86–98. https://doi.org/10.1086/657435

Cassill, D. L. (2019). Extending r/K selection with a maternal risk-management model that classifies animal species into divergent natural selection categories. *Scientific Reports*, *9*(1), 6111. https://doi.org/10.1038/s41598-019-42562-7

Chamberlain, S. A., Szöcs, E., Scott Chamberlain, & Eduard Szocs. (2013). taxize: taxonomic search and retrieval in R. *F1000Research*, *2*(1), 191. https://doi.org/10.12688/f1000research.2-191.v2

Clutton-Brock, T. H., Maccoll, A., Chadwick, P., Gaynor, D., Kansky, R., & Skinner, J. D. (1999). Reproduction and survival of suricates (Suricata suricatta) in the southern Kalahari. *African Journal of Ecology*, *37*(1), 69–80. https://doi.org/10.1046/j.1365-2028.1999.00160.x

Cooke, R. S. C., Bates, A. E., & Eigenbrod, F. (2019). Global trade-offs of functional redundancy and functional dispersion for birds and mammals. *Global Ecology and Biogeography*, *28*(4), 484–495. https://doi.org/10.1111/geb.12869

Diniz-Filho, J. A. F., Bini, L. M., Rangel, T. F., Morales-Castilla, I., Olalla-Tárraga, M. Á., Rodríguez, M. Á., & Hawkins, B. A. (2012). On the selection of phylogenetic eigenvectors for ecological analyses. *Ecography*, *35*(3), 239–249. https://doi.org/10.1111/j.1600-0587.2011.06949.x

Diniz Filho, J. A. F., Villalobos, F., & Bini, L. M. (2015). The best of both worlds: Phylogenetic eigenvector regression and mapping. *Genetics and Molecular Biology*, *38*(3), 396–400. https://doi.org/10.1590/S1415-475738320140391

Dobson, F. S., & Oli, M. K. (2007). Fast and slow life histories of mammals. *Ecoscience*, *14*(3), 292–297. https://doi.org/10.2980/1195-6860(2007)14[292:FASLHO]2.0.CO;2

Etard, A., Morrill, S., & Newbold, T. (2020). Global gaps in trait data for terrestrial vertebrates. *Global Ecology and Biogeography*, geb.13184. https://doi.org/10.1111/geb.13184

Fox, J., & Weisberg, S. (2019). *An R Companion to Applied Regression* (third). Thousand Oaks, California: Sage. Retrieved from https://socialsciences.mcmaster.ca/jfox/Books/Companion/

Gonçalves, F., Bovendorp, R. S., Beca, G., Bello, C., Costa-Pereira, R., Muylaert, R. L., … Galetti, M. (2018). ATLANTIC MAMMAL TRAITS: a data set of morphological traits of mammals in the Atlantic Forest of South America. *Ecology*, *99*(2), 498–498. https://doi.org/10.1002/ecy.2106

Grimm, A., Prieto Ramírez, A. M., Moulherat, S., Reynaud, J., Henle, K., Ramírez, A. M. P., … Henle, K. (2014). Life-history trait database of European reptile species. *Nature Conservation*, *9*, 45–67. https://doi.org/10.3897/natureconservation.9.8908

Hastie, T. (2020). gam: Generalized Additive Models. Retrieved from https://cran.r-project.org/package=gam

Healy, K., Ezard, T. H. G., Jones, O. R., Salguero-Gómez, R., & Buckley, Y. M. (2019). Animal life history is shaped by the pace of life and the distribution of age-specific mortality and reproduction. *Nature Ecology and Evolution*, *3*(8), 1217–1224. https://doi.org/10.1038/s41559-019-0938-7

Hedges, S. B., Marin, J., Suleski, M., Paymer, M., & Kumar, S. (2015). Tree of Life Reveals Clock-Like Speciation and Diversification. *Molecular Biology and Evolution*, *32*(4), 835–845. https://doi.org/10.1093/molbev/msv037

Hyndman, R. J., & Koehler, A. B. (2006). Another look at measures of forecast accuracy. *International Journal of Forecasting*, *22*(4), 679–688. https://doi.org/10.1016/j.ijforecast.2006.03.001

Jones, K. E., Bielby, J., Cardillo, M., Fritz, S. A., O’Dell, J., Orme, C. D. L., … Purvis, A. (2009). PanTHERIA: a species-level database of life history, ecology, and geography of extant and recently extinct mammals. *Ecology*, *90*(9), 2648–2648. https://doi.org/10.1890/08-1494.1

Kuznetsova, A., Brockhoff, P. B., & Christensen, R. H. B. (2017). lmerTest Package: Tests in Linear Mixed Effects Models. *Journal of Statistical Software*, *82*(13). https://doi.org/10.18637/jss.v082.i13

Leung, B., Hargreaves, A. L., Greenberg, D. A., McGill, B., Dornelas, M., & Freeman, R. (2020). Clustered versus catastrophic global vertebrate declines. *Nature 2020 588:7837*, *588*(7837), 267–271. https://doi.org/10.1038/s41586-020-2920-6

Li, W., MacBean, N., Ciais, P., Defourny, P., Lamarche, C., Bontemps, S., … Peng, S. (2017). Gross and net land cover changes based on plant functional types derived from the annual ESA CCI land cover maps. *Earth System Science Data Discussions*, 1–23. https://doi.org/10.5194/essd-2017-74

Lobaina, I. (2014). *Evolution of Maternal Investment Strategies for the Order Crocodylia*. University of South Florida St. Petersburg. Retrieved from http://dspace.nelson.usf.edu:8080/xmlui/bitstream/handle/10806/10009/USFSP Honors Thesis - Ileisy Lobaina.pdf?sequence=1

Meiri, S. (2018). Traits of lizards of the world: Variation around a successful evolutionary design. *Global Ecology and Biogeography*, *27*(10), 1168–1172. https://doi.org/10.1111/geb.12773

Michonneau, F., Brown, J. W., & Winter, D. J. (2016). rotl: an R package to interact with the Open Tree of Life data. *Methods in Ecology and Evolution*, *7*(12), 1476–1481. https://doi.org/10.1111/2041-210X.12593

Murali, G., de Oliveira Caetano, G. H., Barki, G., Meiri, S., & Roll, U. (2022). Emphasizing declining populations in the Living Planet Report. *Nature*, *601*(7894), E20–E24. https://doi.org/10.1038/s41586-021-04165-z

Myhrvold, N. P., Baldridge, E., Chan, B., Sivam, D., Freeman, D. L., & Ernest, S. K. M. (2015). An amniote life-history database to perform comparative analyses with birds, mammals, and reptiles. *Ecology*, *96*(11), 3109. https://doi.org/10.1890/15-0846r.1

Novosolov, M., Raia, P., & Meiri, S. (2013). The island syndrome in lizards. *Global Ecology and Biogeography*, *22*(2), 184–191. https://doi.org/10.1111/j.1466-8238.2012.00791.x

Novosolov, M., Rodda, G. H., North, A. C., Butchart, S. H. M., Tallowin, O. J. S., Gainsbury, A. M., & Meiri, S. (2017). Population density–range size relationship revisited. *Global Ecology and Biogeography*, *26*(10), 1088–1097. https://doi.org/10.1111/geb.12617

Pacifici, M., Santini, L., Di Marco, M., Baisero, D., Francucci, L., Marasini, G. G., … Rondinini, C. (2013). Generation length for mammals. *Nature Conservation*, *5*, 87–94. https://doi.org/10.3897/natureconservation.5.5734

Penone, C., Davidson, A. D., Shoemaker, K. T., Di Marco, M., Rondinini, C., Brooks, T. M., … Costa, G. C. (2014). Imputation of missing data in life-history trait datasets: which approach performs the best? *Methods in Ecology and Evolution*, *5*(9), 961–970. https://doi.org/10.1111/2041-210X.12232

Pinheiro, J., Bates, D., DebRoy, S., & R Core Team. (2021). nlme: Linear and Nonlinear Mixed Effects Models. *Version 3.1-157*. Retrieved from https://cran.r-project.org/package=nlme

Polović, L., Pešić, V., Ljubisavljević, K., & Čadenović, N. (2013). Preliminary data on the reproductive characteristics and diet in an insular population of the lacertid lizard Algyroides nigropunctatus. *North-Western Journal of Zoology*, *9*(1), 201–205.

R Core Team. (2021). R: A Language and Environment for Statistical Computing. *R Foundation for Statistical Computing*. Retrieved from https://www.r-project.org/

Read, A. F., & Harvey, P. H. (1989). Life history differences among the eutherian radiations. *Journal of Zoology*, *219*(2), 329–353. https://doi.org/https://doi.org/10.1111/j.1469-7998.1989.tb02584.x

Scharf, I., Feldman, A., Novosolov, M., Pincheira-Donoso, D., Das, I., Böhm, M., … Meiri, S. (2015). Late bloomers and baby boomers: ecological drivers of longevity in squamates and the tuatara. *Global Ecology and Biogeography*, *24*(4), 396–405. https://doi.org/10.1111/geb.12244

Schwarz, R., & Meiri, S. (2017). The fast-slow life-history continuum in insular lizards: a comparison between species with invariant and variable clutch sizes. *Journal of Biogeography*, *44*(12), 2808–2815. https://doi.org/10.1111/jbi.13067

Sherman, P. W., Braude, S., & Jarvis, J. U. M. (1999). Litter Sizes and Mammary Numbers of Naked Mole-Rats: Breaking the One-Half Rule. *Journal of Mammalogy*, *80*(3), 720–733. https://doi.org/10.2307/1383241

Sherman, Paul W., & Jarvis, J. U. M. (2002). Extraordinary life spans of naked mole-rats ( Heterocephalus glaber ). *Journal of Zoology*, *258*(3), 307–311. https://doi.org/10.1017/S0952836902001437

Smith, F. A., Lyons, S. K., Ernest, S. K. M., Jones, K. E., Kaufman, D. M., Dayan, T., … Haskell, J. P. (2003). Body Mass of Late Quaternary Mammals. *Ecology*, *84*(12), 3403–3403. https://doi.org/10.1890/02-9003

Stark, G., Tamar, K., Itescu, Y., Feldman, A., & Meiri, S. (2018). Cold and isolated ectotherms: drivers of reptilian longevity. *Biological Journal of the Linnean Society*, *125*(4), 730–740. https://doi.org/10.1093/biolinnean/bly153

Stearns, S. C. (1983). The Influence of Size and Phylogeny on Patterns of Covariation among Life-History Traits in the Mammals. *Oikos*, *41*(2), 173. https://doi.org/10.2307/3544261

Stekhoven, D. J., Buhlmann, P., & Bühlmann, P. (2012). MissForest--non-parametric missing value imputation for mixed-type data. *Bioinformatics*, *28*(1), 112–118. https://doi.org/10.1093/bioinformatics/btr597

Trochet, A., Moulherat, S., Calvez, O., Stevens, V. M., Clobert, J., & Schmeller, D. S. (2014). A database of life-history traits of European amphibians. *Biodiversity Data Journal*, *2*(1). https://doi.org/10.3897/BDJ.2.e4123

Verde Arregoitia, L. D., Blomberg, S. P., & Fisher, D. O. (2013). Phylogenetic correlates of extinction risk in mammals: Species in older lineages are not at greater risk. *Proceedings of the Royal Society B: Biological Sciences*, *280*(1765). https://doi.org/10.1098/rspb.2013.1092

Wilman, H., Belmaker, J., Simpson, J., de la Rosa, C., Rivadeneira, M. M., & Jetz, W. (2014). EltonTraits 1.0: Species-level foraging attributes of the world’s birds and mammals. *Ecology*, *95*(7), 2027–2027. https://doi.org/10.1890/13-1917.1

Zuur, A. F., Ieno, E. N., Walker, N., Saveliev, A. A., & Smith, G. M. (2009). *Mixed effects models and extensions in ecology with R*. *Public Health* (1st ed., Vol. 36). New York, NY: Springer New York. https://doi.org/10.1007/978-0-387-87458-6
